# Supplementary material for: Exploring preventive practices of newborn hypothermia among post-natal mothers in selected public health facilities in Kilimanjaro region: Qualitative descriptive study
Source: PLoS One. 2026 Sep 10;21(9):e0357756. doi: 10.1371/journal.pone.0357756 (PMC13561377; doi:10.1371/journal.pone.0357756)
Supplement: Supplementary file 3 [file pone.0357756.s001.docx]

Interview guide

**Guide Questions**

**Topics**

QN1: In your opinion, should neonates be kept warm after birth? Probe: Why? Is it possible to detect hypothermia after delivery to your neonates? How is it done? Have you performed, and how did you do it?

QN2: How do you keep your neonates warm after delivery? At home?  Probe; skin-to-skin contact, breastfeeding,

QN3: Can you share the challenges of keeping your neonates warm, how you cope with them,

Probe: Any support from family, community, and healthcare providers?
