# Supplementary material for: Exploring preventive practices of newborn hypothermia among post-natal mothers in selected public health facilities in Kilimanjaro region: Qualitative descriptive study
Source: PLoS One. 2026 Sep 10;21(9):e0357756. doi: 10.1371/journal.pone.0357756 (PMC13561377; doi:10.1371/journal.pone.0357756)
Supplement: Supplementary file 10002 [file pone.0357756.s003.pdf]

# PLOS One

## Exploring preventive practices of newborn hypothermia among post-natal mothers in selected public health facilities in Kilimanjaro region: Qualitative descriptive study

--Manuscript Draft--

| | |
|---|---|
| **Manuscript Number:** | PONE-D-25-62885R1 |
| **Article Type:** | Research Article |
| **Full Title:** | Exploring preventive practices of newborn hypothermia among post-natal mothers in selected public health facilities in Kilimanjaro region: Qualitative descriptive study |
| **Short Title:** | Preventive practices of newborn hypothermia among post natal mothers |
| **Corresponding Author:** | Emmanuel Daniel, MSc. Midwifery<br>Kilimanjaro college of health and allied sciences<br>Dodoma, TANZANIA, UNITED REPUBLIC OF |
| **Keywords:** | Post natal mothers, preventive practices, newborn hypothermia and thermal care |
| **Abstract:** | Abstract<br>Background<br>Newborn hypothermia is a condition that affects newborn survival if measures are not taken to prevent heat loss, especially in the first 28 days of life. Various studies have been conducted in this area, but there is a lack of information about how post-natal mothers specifically keep their newborns warm in our setting. This study explored the preventive practices for newborn hypothermia among post-natal mothers in selected health facilities in Kilimanjaro region.<br>Method<br>A qualitative descriptive study with a constructivist approach was conducted from April to June 2024 in the Kilimanjaro region involving post-natal mothers. Participants were selected purposively at the post-natal ward. In-depth interview, guided by semi semi-structured interview guide developed by researchers together with an audio recorder, was used to collect information from the participants. Data was analyzed using conventional thematic analysis.<br>Results<br>Twelve (12) semi-structured interviews were conducted with post-natal mothers. Four (4) themes and eight sub-themes emerged; Body covering measures (Covering baby with multiple coats of cottoned materials, Preparing baby clothing before delivery), diversity of room temperature controlling measures(Maintaining indoor heating to warm the room and maintaining indoor lighting to increase room temperature),  skin-to-skin and feeding practices(Keep a baby in physical contact with the mother for warmth and Breastfeeding practices and body caring measures(Massaging the neonates with oil to maintain skin warmth and bathing practices).<br>Conclusion<br>Effective teaching education needs to be addressed to post-natal mothers during antenatal contacts and post-natal check up on good preventive practices of newborn hypothermia to promote a positive behavior on thermal newborn protection. |
| **Order of Authors:** | Emmanuel Daniel, MSc. Midwifery |
| | Saada A Seif, PhD |
| | Walter C Millanzi, PhD |
| **Response to Reviewers:** | Tittle: Exploring preventive practices of newborn hypothermia among post-natal mothers in selected public health facilities in Kilimanjaro region: Qualitative descriptive study<br>Ref: Submission ID: PONE-D-25-62885<br><br>JOURNAL REQUIREMENT<br>1When submitting your revision, we need you to address these additional requirements. 1. Please ensure that your manuscript meets PLOS ONE's style requirements, including those for file naming. The PLOS ONE style templates can be found at<br>https://journals.plos.org/plosone/s/file?id=wjVg/PLOSOne_formatting_sample_main_bo |

Powered by Editorial Manager® and ProduXion Manager® from Aries Systems Corporation

dy.pdf and
https://journals.plos.org/plosone/s/file?id=ba62/PLOSOne_formatting_sample_title_authors_affiliations.pdf
Response: Thank you. We have made sure that our manuscript to abide with PLOS ONE style requirement
2Your ethics statement should only appear in the Methods section of your manuscript. If your ethics statement is written in any section besides the Methods, please delete it from any other section.
Response: Thank you for the valuable comment. The ethical statement has been included in the method section
3In the online submission form, you indicated that all data will be available from the corresponding author upon request.
Response: Concerning the data set of the underlying results of the manuscript. All relevant data are within the manuscript and its supporting files. Raw data, such as transcripts and translations, cannot be shared publicly because of the confidentiality assurance given to participants at the time of consent. Hence, the data can be accessed through the institutional research review committee (IRRC) of the University of Dodoma through drpc@udom.ac.tz after being processed and accepted by the committee.

4Please amend your authorship list in your manuscript file to include author Emmanuel Daniel.
Response: Author Emmanuel Daniel is included in the revised manuscript.

5Please amend the manuscript submission data (via Edit Submission) to include author Emmanuel Daniel Kija.
Response: Author Emmanuel Daniel Kija is omitted in the submission and in the manuscript.
6. Please remove all personal information, ensure that the data shared are in accordance with participant consent, and re upload a fully anonymized data set. Note: spreadsheet columns with personal
Response: All person information is removed in the revised manuscript
7Please include captions for your supporting information files at the end of your manuscript, and update any in-text citations to match accordingly. Please see our Supporting Information
Response: Thank you, caption for supporting information file is included in text citatio

REVIEWER #1: AUTHOR'S RESPONSES TO COMMENTS
S/NREVIEWER'S COMMENTAUTHOR'S RESPONSE
1Why were the demographic information of the two participants interviewed to ensure data adequacy not reported? And were the interviews transcribed and analyzed? Please explain in detail and if not included in the analysis, amend this section of the results and explicitly state in the limitations section that further interviews were not conducted to ensure data adequacy after saturation was reached
Response: Thank you for your constructive comments. Amendments have been made to comply with your comment Page 10, 26 and 27
2Add a column in Table 2 and report the open codes for each subtheme to facilitate tracking of findings and provide comprehensive findings for future researchers.
Response: Column in table 2 has been added to include open codes from participants quotes Page 13 -15
3What is described in the text on line 403 for the number of subthemes in theme " " does not match the number of subthemes in the rest of the text and in Table 2. Please review and correct.
Response: Thank you for the constructive comment. The comment has been addressed in the revised manuscript Pages 21
4Undoubtedly, the limitations of the study, especially methodological ones, include more issues that researchers need to pay attention to and carefully point out, including the use of only mothers' opinions and the failure to address the experiences of health professionals such as midwives and pediatricians who interacted with these mothers and infants and who have shared experiences. Meanwhile, triangulation was also created at the data source level, adding to the strength of the study
Response: The limitations of the study have been revised and now include the suggestion that the study relied on mothers' opinions and failed to account for the

Powered by Editorial Manager® and ProduXion Manager® from Aries Systems Corporation

| | |
|---|---|
| | experiences of health professionals, such as midwives and paediatricians. Pages 26 and 27<br><br><br>REVIEWER #2: AUTHOR'S RESPONSES TO COMMENTS<br>S/NREVIEWER'S COMMENTAUTHOR'S RESPONSE<br>1Please make the manuscript region-specific. There is a difference in the care provided in the high-, middle- and low-income countries.<br>• Add these parameters in the introduction, discussion and methods as well.<br>Response: Thank you for the constructive comment. The study explored more about lower-middle-income countriesAll manuscript<br>2There is need to make the description of themes in a crisper and shortened format.<br>• The quotes of the patients may also be tabulated along with the themes<br>Response: Thanks for the comment, themes remained as it is short according to our view and  open codes instead of patient quotes have been added in Table 2 to track findings, since patient codes may be tracked in the results Page 13-15<br>3. Our settings should be changed to a hospital setting. and Remove word semi semi-structured duplication<br>Response: Setting has been included in the revised manuscript, and the word semi has been omittedPage 2<br>4.Temperature fluctuation does not lead to asphyxia, and has to be deleted<br>Response: Changes have been made in the revised manuscriptPage 3<br>5. This also shows there was a need to intervene appropriately by providing appropriate thermal care. This would be one of the ways to reduce NMR, not the only one.<br>Response: Thank you for the comment, the sentence has been revised and refinedPage 3<br>6. What is the meaning of double increase rather than decline<br>Response: The meaning of double increase rather than decline means that the newborn mortality is still high compared to the Sustainable Development Goal of reducing newborn mortality to 12 per 1000 live births by 20230, as the newborn mortality rate in Tanzania is 24 per 1000 live births.Page 3<br>7. Ensure uniform citation formatting according to journal requirements<br>Response: All citations have been formatted in the revised manuscript<br>8. Strengthen the "knowledge gap" statement: explicitly indicate what is missing in existing literature from Tanzania/Kilimanjaro.Strengthening the "knowledge gap"<br>Response: Strengthen the "knowledge gap" statement  is done in the revised manuscriptPage 4<br>9. The rationale of the study being carried out in that particular region needs to be tailored according to the region and so needs modification in the wording<br>Response: Modification has been carried out to make it clearPage 4<br>10. The rationale given for the usage of this design may be avoided as it's a known fact.<br>Response: The comment has been incorporated in the revised manuscript Page 5<br>11. Researchers chose the purposeful sampling technique to identify the population likely to possess detailed information on newborn hypothermia preventive practices" introduces a significant bias in the study. The mothers who know how to prevent in a detailed manner will be able to provide biased information.<br>Response: We appreciate your constructive feedback and had added limitation paragraph in the revised manuscript to clearly address this comment Page 26 and 27<br>12. Majority of the women were entrepreneur, employed. This also leads to bias. Describe strategies used to ensure maximum variation in sampling.<br>Response: Thank you for this valid concern about potential selection bias due to the overrepresentation of entrepreneurs and employed women. We incorporated strategies for maximum variation in our study to capture diverse socioeconomic and occupational perspectives through stratification of participants by contextual and key demographic characteristics to ensure heterogeneity beyond employment status, such as parity, education level, facility level, and experiences Page 6 and 12<br>13. Mention if the interview guide was pilot-tested or iteratively modified.<br>Response: Thank you for the valid comment, the tool was piloted and details has been included in revised manuscriptPage 8<br>14. Add contextual details of the facilities(rural/urban, patient flow, type of maternity services). • Explain why these specific facilities were chosen<br>Response: Thanks for the comment, contextual details of the facilities has been added |

| | |
|---|---|
| | in the revised manuscriptPage 6<br>15. Describe how coding disagreements were resolved.<br>Response: Coding disagreements were reviewed and discussed by the research team as explained in the revised manuscriptsPage 10<br>15. Mention number of participants contributing to each theme (COREQ requirement).<br>Response: Thank you for the constructive comment. An explanation about the number of participants contributing to each theme is added in the revised manuscriptPage 9<br>16. Add a short subsection on the study’s strengths (currently absent).<br>Response: Subsection of strength has been added in the current manuscriptsPage 26<br>17. Provide a clearer articulation of how this work adds to regional evidence.<br>Response: Articulation of how this works adds to regional evidence has been added in the revised manuscriptPage 26<br>18. References need rewriting.<br>Response: Thank you for the constructive feedback, all references have been formatted again in the revised manuscriptPage 29,30,31 and 32 |
| **Additional Information:** | |
| **Question** | **Response** |
| **Financial Disclosure**<br><br>Enter a financial disclosure statement that describes the sources of funding for the work included in this submission. Review the submission guidelines for detailed requirements. View published research articles from *PLOS ONE* for specific examples.<br><br>This statement is required for submission and **will appear in the published article** if the submission is accepted. Please make sure it is accurate.<br><br>**Funded studies**<br>Enter a statement with the following details:<br>• Initials of the authors who received each award<br>• Grant numbers awarded to each author<br>• The full name of each funder<br>• URL of each funder website<br>• Did the sponsors or funders play any role in the study design, data collection and analysis, decision to publish, or preparation of the manuscript?<br><br>Did you receive funding for this work? | The author(s) received no specific funding for this work. |
| **Competing Interests**<br><br>Use the instructions below to enter a competing interest statement for this submission. On behalf of all authors, | The authors have declared that no competing interests exist. |

| | |
|---|---|
| disclose any competing interests that could be perceived to bias this work—acknowledging all financial support and any other relevant financial or non-financial competing interests.<br><br>This statement is required for submission and **will appear in the published article** if the submission is accepted. Please make sure it is accurate and that any funding sources listed in your Funding Information later in the submission form are also declared in your Financial Disclosure statement.<br><br>View published research articles from *PLOS ONE* for specific examples.<br><br>**NO authors have competing interests**<br><br>Enter: *The authors have declared that no competing interests exist.*<br><br>**Authors with competing interests**<br><br>Enter competing interest details beginning with this statement:<br><br>*I have read the journal's policy and the authors of this manuscript have the following competing interests: [insert competing interests here]*<br><br>* typeset | |
| **Data Availability**<br><br>Authors are required to make all data underlying the findings described fully available, without restriction, and from the time of publication. PLOS allows rare exceptions to address legal and ethical concerns. See the PLOS Data Policy and FAQ for detailed information.<br><br>A Data Availability Statement describing where the data can be found is required at submission. Your answers to this question | Yes - all data are fully available without restriction |

| | |
|---|---|
| constitute the Data Availability Statement and **will be published in the article**, if accepted.<br><br>**Important:** Stating 'data available on request from the author' is not sufficient. If your data are only available upon request, select 'No' for the first question and explain your exceptional situation in the text box.<br><br>Do the authors confirm that all data underlying the findings described in their manuscript are fully available without restriction? | |
| Additional data availability information: | Tick here if your circumstances are not covered by the questions above and you need the journal's help to make your data available. |
| **Ethics Statement**<br><br>Enter an ethics statement for this submission. This statement is required if the study involved:<br><br>• Human participants<br>• Human specimens or tissue<br>• Vertebrate animals or cephalopods<br>• Vertebrate embryos or tissues<br>• Field research<br><br>Write "N/A" if the submission does not require an ethics statement.<br><br>General guidance is provided below. Consult the submission guidelines for detailed instructions. **Make sure that all information entered here is included in the Methods section of the manuscript.** | Ethical clearance was sought from the institutional research review committee (IRRC) of the University of Dodoma on 30 January 2024, with Ref. No. MA.84/261/69/2. The study abided by the ethical principles of the Declaration of Helsinki by the World Medical Association (2001). The participants, before the data collection process, were given detailed information about the purpose of the study, benefits and risks of participation, research procedures, participants' roles in the study, and freedom to participate freely and withdraw from the study at any time they wish. Each participant was asked to sign an informed consent form before participation. Furthermore, participant anonymity was maintained to ensure the confidentiality and privacy of the participants and all information from the field was secured for authorised persons only. Furthermore, during qualitative data collection, the confidentiality and integrity of the data that were collected through audio recording were guaranteed by the implementation of security measures, such as encoding and password protection. Also, anonymisation and privacy maintenance were adhered to |

Powered by Editorial Manager® and ProduXion Manager® from Aries Systems Corporation

| | |
|---|---|
| **Format for specific study types**<br><br>**Human Subject Research (involving human participants and/or tissue)**<br>• Give the name of the institutional review board or ethics committee that approved the study<br>• Include the approval number and/or a statement indicating approval of this research<br>• Indicate the form of consent obtained (written/oral) or the reason that consent was not obtained (e.g. the data were analyzed anonymously)<br><br>**Animal Research (involving vertebrate animals, embryos or tissues)**<br>• Provide the name of the Institutional Animal Care and Use Committee (IACUC) or other relevant ethics board that reviewed the study protocol, and indicate whether they approved this research or granted a formal waiver of ethical approval<br>• Include an approval number if one was obtained<br>• If the study involved *non-human primates*, add *additional details* about animal welfare and steps taken to ameliorate suffering<br>• If anesthesia, euthanasia, or any kind of animal sacrifice is part of the study, include briefly which substances and/or methods were applied<br><br>**Field Research**<br><br>Include the following details if this study involves the collection of plant, animal, or other materials from a natural setting:<br>• Field permit number<br>• Name of the institution or relevant body that granted permission | |
| <strong>Data Availability</strong><br><br><br>Before publication, Authors are required to make fully available and without restriction all data underlying their findings. Sharing the data underlying your findings increases the visibility, credibility, and impact of your work, while supporting transparency and future scientific discovery. Please see our <a href="https://journals.plos.org/plosone/s/data-availability" target="_blank">PLOS Data Policy page</a> for detailed information on this policy.</p><br><br>A <strong>Data Availability | Concerning the data set of the underlying results of the manuscript. All relevant data are within the manuscript and its supporting files. Raw data, such as transcripts and translations, cannot be shared publicly because of the confidentiality assurance given to participants at the time of consent. Hence, the data can be accessed through the institutional research review committee (IRRC) of the University of Dodoma through drpc@udom.ac.tz after being processed and accepted by the committee. |

| | |
|---|---|
| Statement</strong>, detailing where the data can be accessed, is required at first submission. Insert your Data Availability Statement in the box below.</p><br><br>Please see the <a href="https://journals.plos.org/plosone/s/submission-guidelines#loc-data-reporting" target="_blank">data reporting</a> of our submission guidelines for instructions on what you need to include in your Data Availability Statement. Examples of acceptable Data Availability Statements are included here for reference:</p><br><ol><br><li>The minimal data set [and accompanying code, where generated] is available at [repository name] via [DOI hyperlink]</li><br><li>No data was generated by this study. The following existing data sources were used: [dataset name] from [dataset location] available via [web address or DOI hyperlink].</li><br><li>For Study Protocols: No datasets were generated or analysed during the current study. All relevant data from this study will be made available upon study completion.</li><br></ol><br><br><i>PLOS allows rare exemptions to address legal and ethical concerns. If you have legal or ethical restrictions, please use the box below to detail these in full sentences for the Journal team to consider.</i></p><br><br>This statement will be typeset if the manuscript is accepted for publication.</p> | |

Powered by Editorial Manager® and ProduXion Manager® from Aries Systems Corporation

Manuscript

Click here to access/download;Manuscript;Emmanuel manuscrip -qualitative.docx

# Exploring preventive practices of newborn hypothermia among post-natal mothers in selected public health facilities in Kilimanjaro region: Qualitative descriptive study

Emmanuel Daniel, BScNE, MSc[1*], Saada A. Seif, BScN, MSc, PhD[2], and Walter C. Millanzi, BScN, MSc, PhD[3]

[1]*Department of Nursing and Midwifery, Kilimanjaro College of Health and Allied Sciences, Moshi, Tanzania

[2] Department of Public Health and Community Health Nursing, School of Nursing, University of Dodoma, Tanzania

[3] Department of Nursing Management and Education, School of Nursing, University of Dodoma, Tanzania

*Corresponding author(s). E-mail(s): kijasawa@gmail.com

**Abstract**

**Background**

Newborn hypothermia is a condition that affects newborn survival if measures are not taken to prevent heat loss, especially in the first 28 days of life. Various studies have been conducted in this area, but there is a lack of information about how post-natal mothers specifically keep their newborns warm in a hospital setting. This study explored the preventive practices for newborn hypothermia among post-natal mothers in selected health facilities in the Kilimanjaro region.

**Method**

A qualitative descriptive study with a constructivist approach was conducted from April to June 2024 in the Kilimanjaro region involving post-natal mothers. Participants were selected purposively at the post-natal ward. In-depth interview, guided by a semi-structured interview guide developed by researchers together with an audio recorder, was used to collect information from the participants. Data was analyzed using conventional thematic analysis.

**Results**

Twelve (12) semi-structured interviews were conducted with post-natal mothers. Four (4) themes and eight sub-themes emerged; Body covering measures (Covering baby with multiple coats of cottoned materials, Preparing baby clothing before delivery), diversity of room temperature controlling measures(Maintaining indoor heating to warm the room and maintaining indoor lighting to increase room temperature), skin-to-skin and feeding practices(Keep a baby in physical contact with the mother for warmth and Breastfeeding practices and body care measures(Massaging the neonates with oil to maintain skin warmth and bathing practices).

## Conclusion

Effective teaching education needs to be addressed to post-natal mothers during antenatal contacts and post-natal check-ups on good preventive practices of newborn hypothermia to promote a positive behavior on thermal newborn protection.

**Keywords**: Postnatal mothers, preventive practices, newborn hypothermia, and thermal care

## Introduction

Every family prepares for a newborn with great anticipation and pleasure, but losing a newborn due to hypothermia, which can be prevented with low-cost resources, can dismantle their family bond and provide psychological trauma. Newborn hypothermia is said to occur when the core body temperature of a newborn drops to 36.5°C and is classified into mild hypothermia ($36^0$C-$36.4^0$C), Moderate hypothermia ($32^0$C-$35.9^0$C), and severe hypothermia <$32^0$C(1).

Newborns are vulnerable to temperature fluctuation, especially in the first 28 days of life, which predisposes them to different complications like hypothermia, hypoglycemia, sepsis, and asphyxia, which are core contributors to newborn mortality(2). According to the World Health Organisation (WHO), it estimated that 2.3 million newborns died in 2022 within 28 days of life, whereby Sub-Saharan countries had the highest newborn mortality in the world at 27 deaths per 1000 live births (6). Moreover, in Tanzania, it is estimated that newborn mortality is about 24 deaths per 1,000 live births, while the target is  12 per 1,000 live births by 2030(3)which indicates a double increase instead of a decline. This also shows there was a need to intervene appropriately by providing appropriate thermal care and other related interventions.

Hypothermia is an important aspect in the avoidance of preventable newborn deaths, so great care is highly required, especially in this period, to achieve Sustainable Development Goal 3.2(4), and post-natal mothers are important in maintaining newborn thermal protection, particularly after being discharged from the hospital when clinical oversight is minimal. Previous studies have shown that most of the post-natal mothers lacked sufficient knowledge on preventing hypothermia(5), some knew exactly how to keep the newborn warm(6), and others had poor practices, like early bathing(7). This also highlights the need to explore the preventive practices of newborn hypothermia.

In lower and middle-income countries(LMIC), the preventive practices employed by post-natal mothers, including skin-to-skin contact, early initiation of breastfeeding, and avoidance of cold exposure, directly impact newborn outcomes(8). Moreover, post-natal mothers play a great role in newborn' lives, and their practices will have an impact on the existence of the problem, as previous studies have reported post-natal mothers did not practice skin-to-skin contact, newborns were soaked in water in the first 24 hours after delivery, and late initiation of breastfeeding was reported, all of which influence newborn hypothermia(9,10). These findings highlight the critical need to explore the preventive practices among postnatal mothers. Moreover, women from different cultures, especially in lower and middle-income countries, have their own way of caring for children to prevent hypothermia; some practices may be detrimental, as they are not scientifically based, such as bathing a newborn within 24 hours after delivery(11,12). Furthermore, there is limited data in the setting on the adopted preventive practices of neonatal hypothermia by postnatal mothers, as previous studies focused on medical treatment and use guidelines rather than exploring the real practices implemented by the postnatal mothers.

Therefore, the study intended to explore the preventive practices of newborn hypothermia among postnatal mothers in the Kilimanjaro region.

## Material and methods

### Study approach and design

This study was grounded in the interpretivist paradigm, adopting a relativist ontology that acknowledges the existence of multiple, subjective realities shaped by individual contexts. To operationalize this perspective, a qualitative descriptive design was employed. Unlike more abstract methodologies, this design was selected for its epistemological ability to provide a thinly theorized yet high-fidelity account of participant's lived experiences. Maintaining a stance of interpretive validity, the researcher sought a direct, robust description of the phenomenon, leveraging the inherent flexibility of the design to capture depth and variations without imposing restrictive, pre-determined theoretical frameworks (13).

### Researcher characteristics and reflexivity

The principal researcher is a midwifery specialist with eight years of clinical and academic experience spanning labor and delivery, postnatal care, and neonatal units. This background provided high levels of theoretical sensitivity, allowing for deep immersion in the clinical stance of newborn care. However, the research recognized that this professional expertise could introduce pre-understandings or subjective biases. To mitigate this, the study adopted a stance of reflexive objectivity. The research employed Husserlian bracketing, deliberately setting aside personal clinical assumptions to approach the data from a tabula rasa perspective. This was operationalized through rigorous reflexive practices, including reflexive journaling, whereas a dedicated log was

maintained to document personal feelings, expectations, and emerging insights, ensuring a clear distinction between participant narratives and researcher interpretations.

Moreover, the study used field notes from the observations of non-verbal cues and environmental context, which were recorded to provide a thick description of the interview setting. Peer debriefing was also employed through ongoing dialogue with the research team, which served as a reflexive check, challenging potential subjectivity and ensuring the findings were anchored strictly in the participants' voices. By utilizing the self as a reflexive instrument rather than a mere data collection tool, the researcher ensured that the findings present an authentic co-construction of meaning, balanced by clinical insight and methodological detachment.

**Study setting**

The study was conducted in selected health facilities, including Hai district hospital, Pasua, Longoi, and Majengo health centres, involving post-natal mothers. These facilities were chosen due to the high number of deliveries and the diversity of care provided to reflect the variation of newborn prevention practices across the Kilimanjaro region. Hai district hospital and Pasua health centre, located in urban areas, handle approximately 236 and 218 deliveries per month, respectively, and offer comprehensive emergency obstetric and newborn care (EmONC), neonatal intensive care units (NICU), as well as basic emergency obstetric and newborn care (BEmonc). Moreover, Majengo and Longoi health centres, situated in urban and rural areas respectively, have around 196 and 60 deliveries each month and provide basic emergency obstetric and newborn care, including kangaroo mother care. Interviews were conducted in private, conducive, and friendly environments within the hospital or health centre.

**Sampling strategy**

The study employed a criterion-based purposive sampling, a strategic approach designed to identify and recruit information-rich cases that align with the research objectives. The primary goal was to engage participants who possessed a broad experiential understanding of the phenomenon, specifically regarding newborn hypothermia preventive practices. To ensure a holistic and comprehensive dataset, the research utilized a maximum variation sampling technique. This involved the deliberate inclusion of participants with diverse socio-demographic backgrounds, including variations in parity, educational attainment, occupational status, and the level of health facility utilized for delivery. This diversity was essential to capture a wide spectrum of perspectives and to mitigate the risk of homogenous bias. In alignment with qualitative methodology, the final sample size was not predetermined. Instead, recruitment was guided by the dual principles of data saturation and information power. Data collection concluded when thematic redundancy was achieved, which was defined as the point where subsequent interviews yielded no novel conceptual insights or emerging themes. Furthermore, the research assessed the adequacy of the data based on the depth and richness of participants' narratives, ensuring that the information power was sufficient to robustly address the research questions.

**Study population**

The study population comprised postpartum mothers with newborns who received delivery services at the selected healthcare facilities. To ensure the phenomenological integrity of the data, participants were selected based on specific inclusion criteria such as being in the postnatal period and possessing the cognitive and linguistic capacity to provide detailed verbal accounts of their experiences. Nevertheless, the study applied strict exclusion criteria to uphold ethical standards and data reliability, including mothers presenting with acute post-delivery complications or those

categorized as critically ill, who were excluded to prioritize maternal well-being and ensure that the participants were in a stable condition to engage in a reflective interview process.

**Data collection method**

A face-to-face in-depth interview was used to collect qualitative data on preventive practices for newborn hypothermia among postnatal mothers.

***Procedure***: The in-depth interview was conducted in a private, comfortable room with adequate lighting where participants felt at ease expressing themselves. To ensure confidentiality and privacy, the door was closed during the interview. The researcher positioned himself directly in front of the participant and introduced himself, along with a research assistant, to establish a therapeutic relationship before beginning. The interviews started after the researcher explained the purpose, benefits, risks, voluntary nature of participation, privacy, and confidentiality. Subsequently, the researcher obtained informed consent from participants and provided codes to protect their identities. Initially, a researcher gathered demographic data and asked participants to consent to an audio recording. The interview was conducted by two researchers: the principal researcher, who led the interview, and a research assistant, a nurse working in the labour and post-natal wards, responsible for noting non-verbal cues and preparing the environment, including setting up and testing the audio recorder. The interview was conducted in Swahili to facilitate active participation.

The researcher began each interview with a general question to gain a broad understanding of the participants' experiences, followed by several probing questions to delve deeper into the preventive practices related to newborn hypothermia. The researcher used an audio recorder to record the interviews and field notes to capture verbal cues and memos. The interview duration depended on

the participant's tolerance and patience in describing their experiences related to the topic being interviewed. However, the duration of the interview varied from 30 to 90 minutes, and the researcher, together with the research assistant, summarised the key issues immediately after each conducted interview.

**Data collection tool**

The qualitative interview guide was used to collect data (S1 File). The researcher designed the tool in accordance with the research question. Before the main data collection, the tool was piloted to 6 participants from a non-study facility to ensure questions were clear, relevant, and culturally acceptable. Their feedback was used to refine the tool accordingly. The tool focused on questions regarding preventive practices of newborn hypothermia with three main interview topics to delve into the details of this phenomenon, enabling participants to share their experiences that closed-ended questions could potentially miss. Additionally, the audio recorder was employed to capture and preserve the audio from the interview. The audio recorder was chosen for its accuracy in recording participant responses, its long-lasting battery life, its large storage capacity, its easy operation, its portability, and its user-friendliness for fieldwork. In addition, the recorder included backup functions to protect against data loss in the event of unanticipated occurrences or technical issues. Furthermore, the audio recorder was pre-tested before the actual data collection to ensure its functionality, data transfer ability, and audible sound capture, thereby ensuring the tool's credibility for this study.

**Data analysis plan**

The study employed Conventional Thematic analysis, following the rigorous six-phase recursive framework established by Braun and Clarke (14). Data preparation began with the verbatim

transcription of audio recordings, followed by a forward- and backward translation process to ensure linguistic equivalence and conceptual density. To enhance the inductive nature of the study, analysis was conducted at the manifest level, prioritizing the explicit meanings shared by participants. The process was operationalized as follows:

Familiarisation was the first stage at the manifest level of thematic analysis that involved transcribing the data from audio to text, reading and re-reading the data, and identifying the initial ideas about the interviewed topics to familiarise with the collected data.

Secondly, generating initial codes was the second stage after familiarisation, where the researcher systematically coded the interesting and peculiar characteristics of the entire data set, assembling data relevant to each code. The researcher identified meaningful units of text in the form of phrases that were highlighted with colours. Moreover, the two coders discussed differences in their codes until they came to a consensus in order to guarantee intercoder reliability.

Thirdly, searching for themes which involved organizing codes from various interview participants into potential themes, combining multiple codes to create a single theme. The researcher identified patterns among the concepts and began to develop themes according to the coding patterns.

Reviewing themes was another stage in which the researcher reviewed and refined the themes to ensure they were accurate and relevant to the data. The themes were compared with the data to ensure a true presentation of the participant's voice.

After identifying the final themes, the researcher defined and named them in a way that presented the real meaning of the phenomenon. Also, the researcher examined the names of the themes to

ensure they were sufficiently descriptive and brief for inclusion in the report. Also, the identified themes were reviewed and discussed by the research team.

Lastly, producing a report and writing up was the final stage of thematic analysis that involved writing the report using the analysed data. The report comprised a narrative description and quotations from participants.

**Trustworthiness**

To ensure the trustworthiness and authenticity of the findings, the study adhered to the criteria proposed by Lincoln and Guba (15), including:

**Credibility:** Credibility was ensured through persistent observation and prolonged engagement of the participants to identify participants' cues, behaviors, and unique features presented by the participants. Additionally, members checked, and triangulation data from participants of different backgrounds, audio recordings, and field notes were used for triangulation, and the interviews were audio recorded and then transcribed verbatim to ensure that each detailed piece of information provided by the participants was captured and analysed.

**Dependability:** Dependability was ensured by recruiting participants purposefully, using an interview guide, and triangulating two methods of data collection using field notes and audio recording, to capture the data during data analysis. Also, audit trails were ensured through a clear explanation of the research process, data collection and analysis to enable external auditors to value the study.

**Confirmability:** This was ensured by participant validation of transcripts (member checking), use of participants' verbatim quotes to present the results, repeated reading of transcripts to grasp the content, and careful generation of themes to reflect participants' voices.

**Transferability:** This was ensured by describing the study setting and participant characteristics that will represent a general population, and by ensuring that the study setting, participant characteristics. Sub-themes and themes obtained from these findings will apply to another similar study setting.

**Ethical consideration**

Ethical clearance was sought from the institutional research review committee (IRRC) of the University of Dodoma on 30 January 2024, with Ref. No. MA.84/261/69/2. The study abided by the ethical principles of the Declaration of Helsinki by the World Medical Association (2001). The participants, before the data collection process, were given detailed information about the purpose of the study, benefits and risks of participation, research procedures, participants' roles in the study, and freedom to participate freely and withdraw from the study at any time they wish. Each participant was asked to sign an informed consent form before participation. Furthermore, participant anonymity was maintained to ensure the confidentiality and privacy of the participants, and all information from the field was secured for authorised persons only. Furthermore, during qualitative data collection, the confidentiality and integrity of the data that were collected through audio recording were guaranteed by the implementation of security measures, such as encoding and password protection. Also, anonymisation and privacy maintenance were adhered to.

# Results

## Participants' social demographic characteristics

Fourteen (14) participants were interviewed, and two (2) post-natal mothers didn't complete the interview. Thematic data saturation was achieved with the 12$^{th}$ interviews. The characteristics of the participants involved in the study are presented in Table 1.

**Table 1: Demographical characteristics of the participants (N=12)**

| ID | Age (years) | Health facility | Marital status | Level of education | Residence | Occupation | Parity |
|---|---|---|---|---|---|---|---|
| **P1** | 28 | Pasua | Married | Higher education | Rural | Entrepreneur | Multiparous |
| **P2** | 26 | Longoi | Married | Primary school | Rural | Entrepreneur | Multiparous |
| **P3** | 26 | Pasua | Married | Secondary school | Rural | Housewife | Multiparous |
| **P4** | 18 | Majengo | Single | Primary school | Rural | Entrepreneur | Primiparous |
| **P5** | 22 | Hai | Married | Secondary school | Urban | Pastoralist | Primiparous |
| **P6** | 31 | Hai | Married | Primary school | Urban | Entrepreneur | Multiparous |
| **P7** | 34 | Longoi | Married | Higher education | Rural | Employed | Multiparous |
| **P8** | 24 | Majengo | Married | Primary school | Urban | Housewife | Multiparous |
| **P9** | 27 | Pasua | Married | Secondary school | Rural | Entrepreneur | Primiparous |
| **P10** | 23 | Pasua | Married | Secondary school | Urban | entrepreneur | Multiparous |
| **P11** | 33 | Hai | Single | Secondary school | Urban | Entrepreneur | Multiparous |
| **P12** | 28 | Longoi | Married | Primary school | Rural | Entrepreneur | Multiparous |

The analysis of the data collected resulted in four themes: body covering measures, diversity of room temperature controlling measures, skin-to-skin and feeding practices, and body care measures. These themes were supported by eight sub-themes which are Covering the baby with

multiple coats of cottoned materials, preparing baby clothing before delivery, maintaining indoor heating to warm the room, maintaining indoor lighting to increase room temperature, keeping a baby in physical contact with the mothers for warmth, breastfeeding practices, Massaging the newborn with oil to maintain skin warmth and Bathing practices as shown in Table 2.

**Table 2: Indicates how themes were developed from sub-themes and codes**

| **Open Codes** | **SUB-THEMES** | **THEME** |
|---|---|---|
| • Covering the baby well with heavy Clothes<br>• Covering the baby with clothes made up of cotton materials that do not permit cold<br>• Dressing a baby with clothes | Covering the baby with multiple coats of cotton materials | Body covering measures |
| • Preparing baby Clothes before coming for delivery.<br>• Preparing baby wearing like caps, socks, before the birth.<br>• Ironing and packing a baby clothes in a beg | Preparing baby clothing before delivery | |
| • Closing windows and doors to keep the room warm.<br>• Blocking draft.<br>• Switching off the fan to prevent cold in the room. | Maintaining indoor heating to warm the room | Diversity of room temperature controlling measures |

| | | |
|---|---|---|
| • Leaving the electric light on to generate heat in the room.<br>• Opening of curtains during the daytime. | Maintaining indoor lighting to increase the room temperature | |
| • Putting the baby into the chest, he gets the heat from the mother.<br>• Sharing body heat by sleeping together. | Keep a baby in physical contact with the mother for warmth | Skin-to-skin and feeding practices |
| • Early breastfeeding can help the child to warm up.<br>• Start breastfeeding within one hour after delivery.<br>• Frequent breastfeeding helps the baby become healthier. | Breastfeeding practices | |
| • Use of curd oil to soften baby skin.<br>• Warm the coconut oil in your hands before the massage.<br>• Massaging the baby's back and legs with oils. | Massaging the newborn with oil to maintain skin warmth | Body care measures |
| • Use of lukewarm water for wiping and bathing the baby.<br>• Bath the baby in a warm room during the daytime. | Bathing practices | |

| | | |
|---|---|---|
| • Pre- warming of baby clothes before bathing | | |

**Theme one: Body covering measures**

Body covering measures refer to all actions that are taken by post-natal mothers to dress their newborn to protect them against cold, these measures include the use of heavy clothes made up of cotton materials, baby shawls, hats, and socks. Participants demonstrated that keeping the baby warm and preventing catching a cold is enhanced by body covering, which will help to maintain the baby's body temperature. Within this theme, two sub-themes were identified.

**i. Covering the baby with multiple coats of cotton materials**

The participants viewed the use of multiple coats of cottoned materials to dress the baby as one of the preventive practices that protects newborns from catching a cold and finally developing hypothermia. These multiple coats of cottoned materials, such as heavy clothes like kitenge and Khanga (a brightly coloured, thick and thin, rectangular cloth), are mostly used by the mothers to dress their newborn, especially those that are made up of cotton since they can retain warmth as compared to non-cotton materials. Some participants described that these materials were prepared before delivery and are used to swaddle the baby from head to lower extremities to protect most of the areas that are sensitive to cold. The quote below from one of the participants attests this;

> *"First of all, you will cover the baby well with heavy clothes that do not permit the cold and which are not wet to free him from the cold" (P7-L)*

Another participant reported that, after the baby is born, it is very important to dress the baby in clothes that are made up of cotton as they do not permit cold, and the following quotes from the participants can justify this.

*"Some clothes are made of materials that aren't able to maintain warmth, which can cause the baby to catch a cold once you cover him. But other clothes are made of cotton materials that can support warmth" (P5-H*)

Furthermore, participants also demonstrated that they cover newborns with many clothes to protect against diseases like pneumonia; however, this justifies their awareness of the complications related to hypothermia if left unattended. A P8-P delivered to her second baby attested.

*"When the baby is born, I put him in socks, a hat, and a lot of wraps so that he doesn't get cold and become warm and prevent him from getting pneumonia" (P8-M)*

**ii. Preparing baby clothing before delivery**

Baby clothing refers to all baby materials that are used to protect the baby against hypothermia. These are widely used by many women who were interviewed, and most of the women demonstrated that before delivery, they make preparation, which involves buying, washing, ironing, and packing them in a prepared bag ready to wait for the expected day for delivery. One of the participants from the interview explains this.

*"Before I came to give birth, I made preparations for baby clothes like caps and socks; I bought, washed, and ironed them. This is because we were also taught during the clinic by nurses to prepare them to protect babies from colds after birth" (P1-P)*

However, it was noted that during the interview, some of the postnatal mothers described that they made preparations for their babies' clothing before delivery, and some didn't prepare them until

they delivered, with the reason that they didn't prepare because they were not sure if they would deliver a live baby. One of the participants reported this

> "*These baby clothes were prepared by others; I did not prepare them. What happens if you make plans but end up not delivering the baby alive? Who is going to dress them?" (P4-M)*

**Theme two: Diversity of room temperature controlling measures**

During an interview, the participants highlighted that to keep the baby warm, there is a need to control the external environment and keep the room warm including closing the window and doors, switching off the fan if available, using of charcoal stove to heat the room and switching on the electric bulb in the room throughout the night with the hopes, these measures can keep the room warm and finally will prevent the baby from catching cold hence preventing hypothermia. From this theme, three sub-themes were developed.

**i. Maintaining indoor heating to warm the room**

Unwanted air current from entering the room is a key challenge that predisposes newborns to hypothermia. To prevent this, the majority of the postnatal mother explained that when they are at home, they close windows, and curtains and switch off the fan to maintain the warmth of the room. Furthermore, other participants use the charcoal stove to warm the room. This practice they do, especially during the winter period, by placing a charcoal stove far away from the bed, either near the door or in the living room, and the heat that will be generated will spread to all rooms and finally to the place where the baby is sleeping. Despite being aware of the consequences of using the charcoal stove in the room, the participants who were interviewed still use it as a preventive practice for newborn hypothermia. As one mother reported:

> “*As for the house, it depends on the environment where you live. Maybe the house has big windows that let in the cold. You need to make a lot of effort to keep the curtains closed to make sure that any part does not let in the cold” (P4-M)*

Another participant said that:

> *“When I return home, I have to close the windows and doors, and I have to switch off the fan while at home” (P3-P)*

Another mother said

> *“Right now, I am a parent, and the room I am going to use should be warm all the time that heat helps the baby, and me too……… I used to light the charcoal stove to boil the soup and water, so the heat would spread in the room. In many cases, the charcoal stove is also harmful; for that reason, I always put it in the living room, and its heat will spread throughout the house, so the child gets air and heat too, because let's not just say heat, even air has its benefits” (P10-P)*

**ii. Maintaining indoor lighting to increase room temperature**

Indoor lighting plays a great role in maintaining the room temperature through heat generation as adequate lighting contributes to an indoor temperature which helps to warm the room and prevent the occurrence of hypothermia among newborns. One of the participants reported that they use electric bulbs or solar bulbs that they switch on throughout the night to maintain the room temperature. This practice prevents newborn hypothermia.

The participant stated that:

> *"Also, switching on an electric light bulb throughout the night, even if you use solar light, helps to increase the temperature in the room" (P7-L)*

Another participant reported that:

> *"In the room, I will make sure to close the windows, the room should not have a fan, and the electric light should be left on so that it can help generate heat, when the baby sleeps, the lights should be left on, like electric bulbs at night" (P12-L)*

**Theme three: Skin-to-skin and feeding practices**

The interview revealed that postnatal mothers use skin-to-skin and feeding practices as a preventive measure against newborn hypothermia. Other participants mentioned that after the baby was delivered, the baby was placed on their chests by the health care providers, and also asked to start breastfeeding immediately as a way of creating a bond with their babies as well as protecting them from getting hypoglycemia, which will lead to hypothermia. Skin to skin is not commonly practised among mothers since few participants mentioned it as a way of preventing newborn hypothermia some of the participants further mentioned that after delivery, they don't practice skin-to-skin contact at their practices because the cord stump is still wet and intact; maybe if the cord drops off also, they said skin-to-skin is for low-birth-weight babies and not for the full term, and also the initiation of breastfeeding was determined by the condition of the baby, such as crying, sleep, mothers' activities after delivery, and early handling of the baby to them from health care providers. Hence, skin-to-skin contact and feeding practices enable the baby to get heat through either flow of the mother's heat to the baby through skin-to-skin contact and also through the milk

that breastfeeding contains a lot of calories, which also is the source of heat. The three sub-themes were identified under this theme.

**i. Keep a baby in physical contact with the mother for warmth**

The narratives show that physical contact, like putting the baby on the mother's chest, is non-existent to almost all of the participants, with a few mothers who were able to mention and practice, especially after delivery. Most of this physical closeness is done after the second stage of labour, when the baby is delivered, the baby is placed on the mother's chest by the health care professional to gain warmth from the mother, but not a regular practice for these post-natal mothers. Furthermore, other mothers demonstrate that putting the baby under the mother's chest is for low-birth-weight babies and not for babies with normal birth weight.

The above statement was explained by the following participant

> *"I also know that when a baby is underweight, he is placed in a kangaroo to get the warmth of his/her mother, but I do not know how it increases the temperature, but I only know that it is for babies with low weight" (P1-P)*

Another participant explained that

> *"Putting the baby on the chest, skin to skin was done after giving birth, the baby was placed after I did not put him again and I have never done that again even with other children" (P7-L)*

Furthermore, other mothers are afraid to have physical closeness with their babies because the stump is still fresh, and physical contact with the baby's abdomen will hurt the stump. This can be justified by the following statement from one of the participants

*At the moment, I can't because I see that the umbilical cord has not been dropped off. On my side, maybe what I can do is, when the cord is dropped off, it will be fine. Maybe I can hurt the umbilical cord of the baby. I think that is the main reason why you cannot put the baby on the chest because the umbilical cord has not dropped off (P12-L).*

**ii. Breastfeeding practices**

Also, breastfeeding practice was mentioned by the mother as a way of preventing newborn hypothermia among newborns. One mother said during feeding, the baby is kept near the mother's abdomen, hence there is a transfer of heat from the mother to the baby, which also helps to keep the baby warm.

*"Also, the breastfeeding method helps because I am warm here and my milk is also warm, so when I breastfeed the baby, the breast milk is going to reduce the cold" (P11-H)*

Another mother said

*The early breastfeeding method can help the child to warm up because when you breastfeed, you hug him, and hence, he gets his mother's warmth (P6-H)*

Moreover, mothers do not perceive breastfeeding as a measure that should be carried out in a rigid schedule and are therefore oriented on feeding on demand, such as crying of the babies, handling of the baby by health care providers and completion of activities such as bathing carried out by the mothers after delivery

*"…….. What helps me know that he wants to suckle is that you can find him crying because of hunger. When you see him calm, you will know that it was hunger" (P11-H)*

**Theme four: Body care measures**

Body care measures involve bathing activities and topical oil application on the newborn's skin to enhance warmth and promote comfort and sleep. Participants during the interview explained that babies need to receive care after delivery, and one of the cares is the use of oil to keep the skin smooth and warm after wiping or bathing using warm water to remove dirt, to promote a good feeling for the baby, and to enable the baby to sleep well. These measures were described by post-natal mothers aimed at softening the skin and promoting skin warmth, and are done a few hours after delivery. Within this theme, two sub-themes emerge: massaging the newborn with oil to maintain skin warmth and bathing practices.

**i. Massaging the newborn with oil to maintain skin warmth**

Applying oil to baby skin may be beneficial, possibly through physical stimulation while applying the oil, which also improves thermoregulation. Newborn's massage, used for preventive as well as therapeutic processes, participants reported using coconut or parachute oil to improve baby's skin and warmth. As one mother described that

*"I apply coconut oil on the baby to soften her skin and the coconut oil also helps to keep the baby warm" (P9-P)*

Coconut or parachute oil massage is common and all post-natal mothers are reported to engage in this behaviour. Applying oil to the baby's skin after delivery is a common practice that was demonstrated by the mothers. Participants also highlighted the use of coconut oil as a means of

warming the baby. Coconut or parachute oil was used to improve skin condition and to reduce the incidence of hypothermia by reducing trans-epidermal water loss, hence improving thermoregulation.

Another participant reported;

> *"There is also applying coconut oil to the baby; you massage the baby with oil, making the baby feel warm. It's coconut oil. I used to massage the neck, legs, and just the whole body, and when you bathe the baby, you have to massage with oil because when you put the baby in the water, he becomes cold……., so he warms quickly" (P8-M)*

**ii. Bathing practices**

The practice of wiping or bathing newborns within health facilities after birth is prevalent due to cultural and knowledge differences, contradicting the standard care of newborn care. Wiping or bathing of the newborn even before 24 hours is common in our setting, since after delivery, the presence of vernix caseosa and blood on the newborn's skin is perceived as dirty. Therefore, the mothers described using lukewarm water obtained from home and soft clothes obtained from local shops to bathe their babies to promote comfort, smooth sleep, and warmth for the baby.

This statement can be justified by the following participants as reported:

> *"I wiped the baby because I noticed that the blood hadn't been wiped off thoroughly. Maybe he wasn't wiped too much due to his skin being soft, but because he continues to be here, I wiped him again. To wipe the baby, I need a*

*soft cloth and lukewarm water. We usually start to wipe him after a few hours post-delivery—not soon after delivery, but before the cold comes in" (P4-M).*

Another participant explained:

*"In my experience, I bathe the baby with warm water twice a day at noon and before sundown to make it comfortable and so that he can sleep well" (P6-H)*

**Discussion**

The study explored individualised preventive practices for newborn hypothermia, in which 12 post-natal mothers participated, and revealed that different preventive practices and other practices were minimally practised. Interviewed post-natal mothers agree on the importance of thermal care to the newborn from birth to 28 days. The research findings indicate that post-natal mothers take different measures to protect their newborn from getting hypothermia, some of the initiatives are body covering measuring, skin-to-skin and feeding practices, prebonding, diversity of room temperature controlling measures, preparing baby clothes before delivery and body care measures such as the use of swaddling materials like heavy cotton clothes, baby costumes like baby shawl, blankets capes and hat.

But is not apparent all mothers take these measures as required to protect their newborn against catching a cold, some of the preventive practices such as skin-to-skin contact are not well practised and are avoided due to fear of hurting the stump as well as breastfeeding practices are not initiated timely and their initiation is determined by the state of their babies either crying, sleeping and awake. According to the West Guji Zone South Ethiopia study, a cross-sectional study by Wako (9) indicated that drying and wrapping, head covering and early initiation of breastfeeding were common practices while delayed bathing and skin-to-skin contact were infrequently practised(16).

This is also similar to our findings which have indicated some of the preventive practices that are taken into consideration were head covering and wrapping in heavy clothes.

Another study done in South India indicated that rooming in and covering with old cotton clothes was among the preventive practices that were performed by the mothers to protect their newborn against hypothermia, while skin-to-skin care was not common practice, these findings were in alignment with our findings which also revealed room in practices, use of heavy cotton material to cover the babies were among of the findings that were taken into consideration by the postnatal mothers in the study area. Similarly, the findings were also consistent with the study done in Kenyata National Hospital, Kenya, a mixed study on thermoregulation practices among mothers with newborn babies, which indicated that rooming in and the use of warm clothes were among thermoregulation practices to protect their babies against hypothermia(17). Also, these findings are similar to the study conducted in Northern India and Malawi, which also found that community members were not familiar with skin-to-skin care and did not practice it (18,19).

Also, the study findings are similar to the study conducted in Ghana to assess the knowledge and quality of essential newborn care practices in La Dade Kotopon Municipality, which indicated that some practices used in keeping babies warm were keeping close to the mother's skin, wrapping with warm clothes, and bathing of the baby with warm water (20). Our findings were contrasted with the study findings done in Islamabad capital territory in Pakistan that assessed essential newborn care practices in squatters' settlements and that of Kenya and revealed that skin-to-skin contact was practised to more than half of the mothers (57%) within ten minutes of birth(21,22), this indicates that skin to skin contact is an evidence-based intervention for regulation and maintenance of newborn body temperature and is also the catalyst for breastfeeding.

Therefore, this study provides a critical evidence gap in newborn hypothermia prevention practices specific to the Kilimanjaro region, which can be generalised to lower- and middle-income countries, revealing an implementation gap despite thermal care practices and policies established by the World Health Organisation.

**Strengths and limitations of the study**

The study provides first-hand preventive practices of newborn hypothermia among postnatal mothers. Moreover, Triangulation was done at the data source level. The study also has limitations, like the study didn't explore the recommended practices versus those not recommended. Also, further interviews were not conducted to ensure data adequacy after saturation was reached. The study relied on mothers' opinions and failed to account for the experiences of health professionals, such as midwives and paediatricians, and the study did not triangulate data from multiple sources, such as from nurses and midwives, which lead to selection bias.

**Conclusion**

In conclusion, body covering measures, diversity of room temperature controlling measures, skin-to-skin contact and feeding practice, and body care measures were among the preventive practices elucidated by post-natal mothers in the study area. However, some preventive practices, like indoor heating using charcoal stoves to warm the room, could be avoided in order to improve newborn and maternal health. Furthermore, skin-to-skin contact, the commencement of breastfeeding within one hour, and covering newborns with caps and socks are priority practices for newborn hypothermia prevention, requiring suitably tailored behaviour modification interventions among post-natal mothers.

## Recommendations

### For further studies

1. The cause of the lack of skin-to-skin care and delayed initiation of breastfeeding should be further explored for a potential solution
2. Further studies, an observation study should be carried out to observe the real practices of post-natal mothers, like skin-to-skin contact, which will help to quantify these practices

### For action

1. The Ministry of Health should develop multifaceted prevention strategies for newborn hypothermia, which should include community-based intervention, empowering postnatal mothers to adhere to recommended practices, and improving the availability of thermal care equipment in the healthcare system.
2. Post-natal mothers should be trained in thermal care during antenatal care contacts to enhance the development of positive behaviours from conception to post-delivery on thermal care practices.

## Supporting file

S1 File: Interview guide


## Acknowledgement

The research team appreciate the administration of the health facilities where this study was conducted.

## Funding source and conflict of interest

The authors have no funding or conflicts of interest to disclose

**Author contributions**

**Conceptualization**: Emmanuel Daniel, Saada A. Seif, Walter C. Millanzi

**Methodology**: Emmanuel Daniel, Saada A. Seif, Walter C. Millanzi

**Investigation**: Emmanuel Daniel

**Data curation**: Emmanuel Daniel

**Formal analysis**: Emmanuel Daniel, Saada A. Seif, Walter C. Millanzi

**Supervision**: Saada A. Seif, Walter C. Millanzi

**Writing -original draft**: Emmanuel Daniel

**Writing -review & editing**: Emmanuel Daniel, Saada A. Seif, Walter C. Millanzi

## References


1. World Health Organization. World Health Organization- Thermal protection of the newborn: practical guide. Maternal and Newborn Health/ Safe Motherhood Unit Division of Retroductive Health. 2013;1–68.

2. Sharkh AA, Ighneimat F, Khdour YF, Aladam NY. Factors contributing to neonatal mortality in a tertiary center in south West Bank : A single- center retrospective cohort study. PLOS Glob Public Heal 5(7) e0004885 [Internet]. 2025;1–9. Available from: https://doi.org/10.1371/journal.pgph.0004885

3. TDHS. Tanzania demographic and health survey [Internet]. TDHS. 2022. Available from: https://dhsprogram.com/pubs/pdf/FR382/FR382.pdf

4. Nations U. The Sustainable Development Goals Report 2019. United Nations Publ issued by Dep Econ Soc Aff [Internet]. 2019;64. Available from: https://unstats.un.org/sdgs/report/2022/%0Ahttps://www.un-ilibrary.org/content/books/9789210018098%0Ahttps://www.un-ilibrary.org/content/books/9789210478878

5. Shah I, Paudyal L, Soti H. Descriptive Cross-Sectional Study on Mother ' S Knowledge. J Univers Coll Med Sci [Internet]. 2022;10(01):62–6. Available from: https://doi.org/10.3126/jucms.v10i01.47244

6. Srivastava A, Saxena S, Srivastava P, Mahmood SE, Pandey R, Saxena A. Newborn thermal care in western Uttar Pradesh — gap analysis between knowledge and practices. Arch Public Heal [Internet]. 2022;1–10. Available from: https://doi.org/10.1186/s13690-022-00809-2

7. Fenta Kebede B, Dagnaw Genie Y, Yetwale Hiwot A, Biyazin T, Abebe B. Early Newborn Bath Practice and Its Associated Factors in Jimma, South West Ethiopia, 2021. Pediatr Heal Med Ther. 2022;Volume 13:43–52.

8. Demtse AG, Pfister RE, Nigussie AK, McClure EM, Ferede YG, Tazu Bonger Z, et al. Hypothermia in Preterm Newborns: Impact on Survival. Glob Pediatr Heal [Internet]. 2020;7. Available from: https://doi.org/10.1177/2333794X20957655

9. Wako WG, Beyene BN, Wayessa ZJ, Fikrie A, Amaje E. Assessment of neonatal thermal cares: Practices and beliefs among rural women in West Guji Zone, South Ethiopia: A cross-sectional study. PLOS Glob Public Heal [Internet]. 2022;2(6):e0000568. Available from: http://dx.doi.org/10.1371/journal.pgph.0000568

10. Memon J, Holakouie-Naieni K, Majdzadeh R, Yekaninejad MS, Garmaroudi G, Raza O, et al. Knowledge, attitude, and practice among mothers about newborn care in Sindh, Pakistan. BMC Pregnancy Childbirth [Internet]. 2019;19(1):1–9. Available from: https://link.springer.com/article/10.1186/s12884-019-2479-0

11. Tulelo PM, Mulaudzi FM. Indigenous neonatal feeding and bathing practices of caregivers in Vhembe district, Limpopo Province. Heal SA Gesondheid [Internet]. 2021;26:1–8. Available from: https://pmc.ncbi.nlm.nih.gov/articles/PMC8661288/

12. Khan SM, Timothy E, Singh K, Carvajal- L. Thermal care of newborns : drying and bathing practices in Malawi and Bangladesh. J Glob Health [Internet]. 2018;8(1):1–10. Available from: https://pmc.ncbi.nlm.nih.gov/articles/PMC5943716/

13. Nowell LS, Norris JM, White DE, Moules NJ. Thematic Analysis: Striving to Meet the Trustworthiness Criteria. Int J Qual Methods. 2017;16(1):1–13.

14. Braun V, Clarke V. Using thematic analysis in psychology. Qual Res Psychol. 2006;3(2):77–101.

15. Brannen J. Mixing methods: The entry of qualitative and quantitative approaches into the research process. Int J Soc Res Methodol Theory Pract. 2005;8(3):173–84.

16. Sjömar J, Ottesen H, Banik G, Rahman AE, Thernström Blomqvist Y, Rahman SM, et al. Exploring caregivers' experiences of Kangaroo Mother Care in Bangladesh: A descriptive qualitative study. PLoS One [Internet]. 2023;18(1 January):1–16. Available from: https://journals.plos.org/plosone/article?id=10.1371/journal.pone.0280254

17. Wanjiru JW, Makworo D, Simba JM. Thermoregulation Practices among Mothers with New-Born Babies Attending Kenyatta National Hospital, Kenya. Galore Int J Heal Sci Res [Internet]. 2021;6(3):82–9.

18. Qazi M, Saqib N, Raina R. Knowledge regarding prevention of hypothermia in newborns among mothers in Northern India. Int J Res Med Sci [Internet]. 2019;7(5):1727. Available from: https://pesquisa.bvsalud.org/portal/resource/pt/sea-211466

19. Nyondo-Mipando AL, Kinshella MLW, Hiwa T, Salimu S, Banda M, Vidler M, et al. Mothers' quality of life delivering kangaroo mother care at Malawian hospitals: a qualitative study. Health Qual Life Outcomes [Internet]. 2021;19(1):1–8. Available from: https://doi.org/10.1186/s12955-021-01823-8

20. Ayete-Nyampong J, Udofia EA. Assessment of knowledge and quality of essential newborn care practices in La Dade Kotopon Municipality, Ghana. PLoS One [Internet]. 2020;15(8 August 2020):1–15. Available from: http://dx.doi.org/10.1371/journal.pone.0237820

21. RH H, SH H, TR R, KH H, JB B, FE E. Assessment of Essential Newborn Care Practices in the Squatter Settlements of Islamabad Capital Territory in Pakistan. J Pediatr Child Heal Care [Internet]. 2021;6(2). Available from: https://austinpublishinggroup.com/pediatrics-child-health-care/fulltext/jpchc-v6-id1043.pdf

22. Bhuiya NA, Liu S, Muyodi D, Bucher SL. Feasibility and acceptability of a novel biomedical device to prevent neonatal hypothermia and augment Kangaroo Mother Care in Kenya: Qualitative analysis of focus group discussions and key Informant Interviews. PLOS Glob Public Heal [Internet]. 2024;4(4):1–20. Available from: http://dx.doi.org/10.1371/journal.pgph.0001708

Click here to access/download
**Supporting Information**
Semi structured interview guide.docx

# Exploring preventive practices of newborn hypothermia among post-natal mothers in selected public health facilities in Kilimanjaro region: Qualitative descriptive study

Emmanuel Daniel, BScNE, MSc[1*], Saada A. Seif, BScN, MSc, PhD[2], and Walter C. Millanzi, BScN, MSc, PhD[3]

[1]*Department of Nursing and Midwifery, Kilimanjaro College of Health and Allied Sciences, Moshi, Tanzania

[2] Department of Public Health and Community Health Nursing, School of Nursing, University of Dodoma, Tanzania

[3] Department of Nursing Management and Education, School of Nursing, University of Dodoma, Tanzania

*Corresponding author(s). E-mail(s): kijasawa@gmail.com

## Abstract

### Background

Newborn hypothermia is a condition that affects newborn survival if measures are not taken to prevent heat loss, especially in the first 28 days of life. Various studies have been conducted in this area, but there is a lack of information about how post-natal mothers specifically keep their newborns warm in a hospital setting. This study explored the preventive practices for newborn hypothermia among post-natal mothers in selected health facilities in the Kilimanjaro region.

### Method

A qualitative descriptive study with a constructivist approach was conducted from April to June 2024 in the Kilimanjaro region involving post-natal mothers. Participants were selected purposively at the post-natal ward. In-depth interview, guided by a semi-structured interview guide developed by researchers together with an audio recorder, was used to collect information from the participants. Data was analyzed using conventional thematic analysis.

### Results

Twelve (12) semi-structured interviews were conducted with post-natal mothers. Four (4) themes and eight sub-themes emerged; Body covering measures (Covering baby with multiple coats of cottoned materials, Preparing baby clothing before delivery), diversity of room temperature controlling measures(Maintaining indoor heating to warm the room and maintaining indoor lighting to increase room temperature), skin-to-skin and feeding practices(Keep a baby in physical contact with the mother for warmth and Breastfeeding practices and body care measures(Massaging the neonates with oil to maintain skin warmth and bathing practices).

## Conclusion

Effective teaching education needs to be addressed to post-natal mothers during antenatal contacts and post-natal check-ups on good preventive practices of newborn hypothermia to promote a positive behavior on thermal newborn protection.

**Keywords**: Postnatal mothers, preventive practices, newborn hypothermia, and thermal care

## Introduction

Every family prepares for a newborn with great anticipation and pleasure, but losing a newborn due to hypothermia, which can be prevented with low-cost resources, can dismantle their family bond and provide psychological trauma. Newborn hypothermia is said to occur when the core body temperature of a newborn drops to 36.5°C and is classified into mild hypothermia ($36^0$C-$36.4^0$C), Moderate hypothermia ($32^0$C-$35.9^0$C), and severe hypothermia <$32^0$C(1).

Newborns are vulnerable to temperature fluctuation, especially in the first 28 days of life, which predisposes them to different complications like hypothermia, hypoglycemia, sepsis, and asphyxia, which are core contributors to newborn mortality(2). According to the World Health Organisation (WHO), it estimated that 2.3 million newborns died in 2022 within 28 days of life, whereby Sub-Saharan countries had the highest newborn mortality in the world at 27 deaths per 1000 live births (6). Moreover, in Tanzania, it is estimated that newborn mortality is about 24 deaths per 1,000 live births, while the target is 12 per 1,000 live births by 2030(3), which indicates a double increase

instead of a decline. This also shows there was a need to intervene appropriately by providing appropriate thermal care and other related interventions.

Hypothermia is an important aspect in the avoidance of preventable newborn deaths, so great care is highly required, especially in this period, to achieve Sustainable Development Goal 3.2(4), and post-natal mothers are important in maintaining newborn thermal protection, particularly after being discharged from the hospital when clinical oversight is minimal. Previous studies have shown that most of the post-natal mothers lacked sufficient knowledge on preventing hypothermia(5), some knew exactly how to keep the newborn warm(6), and others had poor practices, like early bathing(7). This also highlights the need to explore the preventive practices of newborn hypothermia.

In lower and middle-income countries(LMIC), the preventive practices employed by post-natal mothers, including skin-to-skin contact, early initiation of breastfeeding, and avoidance of cold exposure, directly impact newborn outcomes(8). Moreover, post-natal mothers play a great role in newborn' lives, and their practices will have an impact on the existence of the problem, as previous studies have reported post-natal mothers did not practice skin-to-skin contact, newborns were soaked in water in the first 24 hours after delivery, and late initiation of breastfeeding was reported, all of which influence newborn hypothermia(9,10). These findings highlight the critical need to explore the preventive practices among postnatal mothers. Moreover, women from different cultures, especially in lower and middle-income countries, have their own way of caring for children to prevent hypothermia; some practices may be detrimental, as they are not scientifically based, such as bathing a newborn within 24 hours after delivery(11,12). Furthermore, there is limited data in the setting on the adopted preventive practices of neonatal hypothermia by postnatal

mothers, as previous studies focused on medical treatment and use guidelines rather than exploring the real practices implemented by the postnatal mothers.

Therefore, the study intended to explore the preventive practices of newborn hypothermia among postnatal mothers in the Kilimanjaro region.

## Material and methods

### Study approach and design

This study was grounded in the interpretivist paradigm, adopting a relativist ontology that acknowledges the existence of multiple, subjective realities shaped by individual contexts. To operationalize this perspective, a qualitative descriptive design was employed. Unlike more abstract methodologies, this design was selected for its epistemological ability to provide a thinly theorized yet high-fidelity account of participant's lived experiences. Maintaining a stance of interpretive validity, the researcher sought a direct, robust description of the phenomenon, leveraging the inherent flexibility of the design to capture depth and variations without imposing restrictive, pre-determined theoretical frameworks (13).

### Researcher characteristics and reflexivity

The principal researcher is a midwifery specialist with eight years of clinical and academic experience spanning labor and delivery, postnatal care, and neonatal units. This background provided high levels of theoretical sensitivity, allowing for deep immersion in the clinical stance of newborn care. However, the research recognized that this professional expertise could introduce pre-understandings or subjective biases. To mitigate this, the study adopted a stance of reflexive objectivity. The research employed Husserlian bracketing, deliberately setting aside personal clinical assumptions to approach the data from a tabula rasa perspective. This was operationalized

through rigorous reflexive practices, including reflexive journaling, whereas a dedicated log was maintained to document personal feelings, expectations, and emerging insights, ensuring a clear distinction between participant narratives and researcher interpretations.

Moreover, the study used field notes from the observations of non-verbal cues and environmental context, which were recorded to provide a thick description of the interview setting. Peer debriefing was also employed through ongoing dialogue with the research team, which served as a reflexive check, challenging potential subjectivity and ensuring the findings were anchored strictly in the participants' voices. By utilizing the self as a reflexive instrument rather than a mere data collection tool, the researcher ensured that the findings present an authentic co-construction of meaning, balanced by clinical insight and methodological detachment.

**Study setting**

The study was conducted in selected health facilities, including Hai district hospital, Pasua, Longoi, and Majengo health centres, involving post-natal mothers. These facilities were chosen due to the high number of deliveries and the diversity of care provided to reflect the variation of newborn prevention practices across the Kilimanjaro region. Hai district hospital and Pasua health centre, located in urban areas, handle approximately 236 and 218 deliveries per month, respectively, and offer comprehensive emergency obstetric and newborn care (EmONC), neonatal intensive care units (NICU), as well as basic emergency obstetric and newborn care (BEmonc). Moreover, Majengo and Longoi health centres, situated in urban and rural areas respectively, have around 196 and 60 deliveries each month and provide basic emergency obstetric and newborn care, including kangaroo mother care. Interviews were conducted in private, conducive, and friendly environments within the hospital or health centre.

**Sampling strategy**

The study employed a criterion-based purposive sampling, a strategic approach designed to identify and recruit information-rich cases that align with the research objectives. The primary goal was to engage participants who possessed a broad experiential understanding of the phenomenon, specifically regarding newborn hypothermia preventive practices. To ensure a holistic and comprehensive dataset, the research utilized a maximum variation sampling technique. This involved the deliberate inclusion of participants with diverse socio-demographic backgrounds, including variations in parity, educational attainment, occupational status, and the level of health facility utilized for delivery. This diversity was essential to capture a wide spectrum of perspectives and to mitigate the risk of homogenous bias. In alignment with qualitative methodology, the final sample size was not predetermined. Instead, recruitment was guided by the dual principles of data saturation and information power. Data collection concluded when thematic redundancy was achieved, which was defined as the point where subsequent interviews yielded no novel conceptual insights or emerging themes. Furthermore, the research assessed the adequacy of the data based on the depth and richness of participants' narratives, ensuring that the information power was sufficient to robustly address the research questions.

**Study population**

The study population comprised postpartum mothers with newborns who received delivery services at the selected healthcare facilities. To ensure the phenomenological integrity of the data, participants were selected based on specific inclusion criteria such as being in the postnatal period and possessing the cognitive and linguistic capacity to provide detailed verbal accounts of their experiences. Nevertheless, the study applied strict exclusion criteria to uphold ethical standards and data reliability, including mothers presenting with acute post-delivery complications or those

categorized as critically ill, who were excluded to prioritize maternal well-being and ensure that the participants were in a stable condition to engage in a reflective interview process.

**Data collection method**

A face-to-face in-depth interview was used to collect qualitative data on preventive practices for newborn hypothermia among postnatal mothers.

***Procedure***: The in-depth interview was conducted in a private, comfortable room with adequate lighting where participants felt at ease expressing themselves. To ensure confidentiality and privacy, the door was closed during the interview. The researcher positioned himself directly in front of the participant and introduced himself, along with a research assistant, to establish a therapeutic relationship before beginning. The interviews started after the researcher explained the purpose, benefits, risks, voluntary nature of participation, privacy, and confidentiality. Subsequently, the researcher obtained informed consent from participants and provided codes to protect their identities. Initially, a researcher gathered demographic data and asked participants to consent to an audio recording. The interview was conducted by two researchers: the principal researcher, who led the interview, and a research assistant, a nurse working in the labour and post-natal wards, responsible for noting non-verbal cues and preparing the environment, including setting up and testing the audio recorder. The interview was conducted in Swahili to facilitate active participation.

The researcher began each interview with a general question to gain a broad understanding of the participants' experiences, followed by several probing questions to delve deeper into the preventive practices related to newborn hypothermia. The researcher used an audio recorder to record the interviews and field notes to capture verbal cues and memos. The interview duration depended on

the participant's tolerance and patience in describing their experiences related to the topic being interviewed. However, the duration of the interview varied from 30 to 90 minutes, and the researcher, together with the research assistant, summarised the key issues immediately after each conducted interview.

## Data collection tool

The qualitative interview guide was used to collect data (S1 File). The researcher designed the tool in accordance with the research question. Before the main data collection, the tool was piloted to 6 participants from a non-study facility to ensure questions were clear, relevant, and culturally acceptable. Their feedback was used to refine the tool accordingly. The tool focused on questions regarding preventive practices of newborn hypothermia with three main interview topics to delve into the details of this phenomenon, enabling participants to share their experiences that closed-ended questions could potentially miss. Additionally, the audio recorder was employed to capture and preserve the audio from the interview. The audio recorder was chosen for its accuracy in recording participant responses, its long-lasting battery life, its large storage capacity, its easy operation, its portability, and its user-friendliness for fieldwork. In addition, the recorder included backup functions to protect against data loss in the event of unanticipated occurrences or technical issues. Furthermore, the audio recorder was pre-tested before the actual data collection to ensure its functionality, data transfer ability, and audible sound capture, thereby ensuring the tool's credibility for this study.

## Data analysis plan

The study employed Conventional Thematic analysis, following the rigorous six-phase recursive framework established by Braun and Clarke (14). Data preparation began with the verbatim

transcription of audio recordings, followed by a forward- and backward translation process to ensure linguistic equivalence and conceptual density. To enhance the inductive nature of the study, analysis was conducted at the manifest level, prioritizing the explicit meanings shared by participants. The process was operationalized as follows:

Familiarisation was the first stage at the manifest level of thematic analysis that involved transcribing the data from audio to text, reading and re-reading the data, and identifying the initial ideas about the interviewed topics to familiarise with the collected data.

Secondly, generating initial codes was the second stage after familiarisation, where the researcher systematically coded the interesting and peculiar characteristics of the entire data set, assembling data relevant to each code. The researcher identified meaningful units of text in the form of phrases that were highlighted with colours. Moreover, the two coders discussed differences in their codes until they came to a consensus in order to guarantee intercoder reliability.

Thirdly, searching for themes which involved organizing codes from various interview participants into potential themes, combining multiple codes to create a single theme. The researcher identified patterns among the concepts and began to develop themes according to the coding patterns.

Reviewing themes was another stage in which the researcher reviewed and refined the themes to ensure they were accurate and relevant to the data. The themes were compared with the data to ensure a true presentation of the participant's voice.

After identifying the final themes, the researcher defined and named them in a way that presented the real meaning of the phenomenon. Also, the researcher examined the names of the themes to

ensure they were sufficiently descriptive and brief for inclusion in the report. Also, the identified themes were reviewed and discussed by the research team.

Lastly, producing a report and writing up was the final stage of thematic analysis that involved writing the report using the analysed data. The report comprised a narrative description and quotations from participants.

**Trustworthiness**

To ensure the trustworthiness and authenticity of the findings, the study adhered to the criteria proposed by Lincoln and Guba (15), including:

**Credibility:**

Credibility was ensured through persistent observation and prolonged engagement of the participants to identify participants' cues, behaviours, and unique features presented by the participants. Additionally, members checked, and triangulation data from participants of different backgrounds, audio recordings, and field notes were used for triangulation, and the interviews were audio recorded and then transcribed verbatim to ensure that each detailed piece of information provided by the participants was captured and analysed.

**Dependability:**

Dependability was ensured by recruiting participants purposefully, using an interview guide, and triangulating two methods of data collection using field notes and audio recording, to capture the data during data analysis. Also, audit trails were ensured through a clear explanation of the research process, data collection and analysis to enable external auditors to value the study.

**Confirmability:**

This was ensured by participant validation of transcripts (member checking), use of participants' verbatim quotes to present the results, repeated reading of transcripts to grasp the content, and careful generation of themes to reflect participants' voices.

**Transferability:**

This was ensured by describing the study setting and participant characteristics that will represent a general population, and by ensuring that the study setting, participant characteristics. Sub-themes and themes obtained from these findings will apply to another similar study setting.

**Ethical consideration**

Ethical clearance was sought from the institutional research review committee (IRRC) of the University of Dodoma on 30 January 2024, with Ref. No. MA.84/261/69/2. The study abided by the ethical principles of the Declaration of Helsinki by the World Medical Association (2001). The participants, before the data collection process, were given detailed information about the purpose of the study, benefits and risks of participation, research procedures, participants' roles in the study, and freedom to participate freely and withdraw from the study at any time they wish. Each participant was asked to sign an informed consent form before participation. Furthermore, participant anonymity was maintained to ensure the confidentiality and privacy of the participants, and all information from the field was secured for authorised persons only. Furthermore, during qualitative data collection, the confidentiality and integrity of the data that were collected through audio recording were guaranteed by the implementation of security measures, such as encoding and password protection. Also, anonymisation and privacy maintenance were adhered to.

# Results

## Participants' social demographic characteristics

Fourteen (14) participants were interviewed, and two (2) post-natal mothers didn't complete the interview. Thematic data saturation was achieved with the 12th interviews. The characteristics of the participants involved in the study are presented in Table 1.

**Table 1: Demographical characteristics of the participants (N=12)**

| ID | Age (years) | Health facility | Marital status | Level of education | Residence | Occupation | Parity |
|---|---|---|---|---|---|---|---|
| **P1** | 28 | Pasua | Married | Higher education | Rural | Entrepreneur | Multiparous |
| **P2** | 26 | Longoi | Married | Primary school | Rural | Entrepreneur | Multiparous |
| **P3** | 26 | Pasua | Married | Secondary school | Rural | Housewife | Multiparous |
| **P4** | 18 | Majengo | Single | Primary school | Rural | Entrepreneur | Primiparous |
| **P5** | 22 | Hai | Married | Secondary school | Urban | Pastoralist | Primiparous |
| **P6** | 31 | Hai | Married | Primary school | Urban | Entrepreneur | Multiparous |
| **P7** | 34 | Longoi | Married | Higher education | Rural | Employed | Multiparous |
| **P8** | 24 | Majengo | Married | Primary school | Urban | Housewife | Multiparous |
| **P9** | 27 | Pasua | Married | Secondary school | Rural | Entrepreneur | Primiparous |
| **P10** | 23 | Pasua | Married | Secondary school | Urban | entrepreneur | Multiparous |

| | | | | | | | |
|---|---|---|---|---|---|---|---|
| **P11** | 33 | Hai | Single | Secondary school | Urban | Entrepreneur | Multiparous |
| **P12** | 28 | Longoi | Married | Primary school | Rural | Entrepreneur | Multiparous |

The analysis of the data collected resulted in four themes: body covering measures, diversity of room temperature controlling measures, skin-to-skin and feeding practices, and body care measures. These themes were supported by eight sub-themes which are Covering the baby with multiple coats of cottoned materials, preparing baby clothing before delivery, maintaining indoor heating to warm the room, maintaining indoor lighting to increase room temperature, keeping a baby in physical contact with the mothers for warmth, breastfeeding practices, Massaging the newborn with oil to maintain skin warmth and Bathing practices as shown in Table 2.

**Table 2: Indicates how themes were developed from sub-themes and codes**

| Open Codes | SUB-THEMES | THEME |
|---|---|---|
| • Covering the baby well with heavy Clothes<br>• Covering the baby with clothes made up of cotton materials that do not permit cold.<br>• Dressing a baby with clothes | Covering the baby with multiple coats of cotton materials | Body covering measures |
| • Preparing baby Clothes before coming for delivery.<br>• Preparing baby wearing like caps, socks, before the birth. | Preparing baby clothing before delivery | |

| | | |
|---|---|---|
| • Ironing and packing a baby clothes in a beg | | |
| • Closing windows and doors to keep the room warm.<br>• Blocking draft.<br>• Switching off the fan to prevent cold in the room. | Maintaining indoor heating to warm the room | Diversity of room temperature controlling measures |
| • Leaving the electric light on to generate heat in the room.<br>• Opening of curtains during the daytime. | Maintaining indoor lighting to increase the room temperature | |
| • Putting the baby into the chest, he gets the heat from the mother.<br>• Sharing body heat by sleeping together. | Keep a baby in physical contact with the mother for warmth | Skin-to-skin and feeding practices |
| • Early breastfeeding can help the child to warm up.<br>• Start breastfeeding within one hour after delivery.<br>• Frequent breastfeeding helps the baby become healthier. | Breastfeeding practices | |
| • Use of curd oil to soften baby skin.<br>• Warm the coconut oil in your hands before the massage. | Massaging the newborn with oil to maintain skin warmth | Body care measures |

| | | |
|---|---|---|
| • Massaging the baby's back and legs with oils. | | |
| • Use of lukewarm water for wiping and bathing the baby.<br>• Bath the baby in a warm room during the daytime.<br>• Pre- warming of baby clothes before bathing | Bathing practices | |

**Theme one: Body covering measures**

Body covering measures refer to all actions that are taken by post-natal mothers to dress their newborn to protect them against cold, these measures include the use of heavy clothes made up of cotton materials, baby shawls, hats, and socks. Participants demonstrated that keeping the baby warm and preventing catching a cold is enhanced by body covering, which will help to maintain the baby's body temperature. Within this theme, two sub-themes were identified.

**i. Covering the baby with multiple coats of cotton materials**

The participants viewed the use of multiple coats of cottoned materials to dress the baby as one of the preventive practices that protects newborns from catching a cold and finally developing hypothermia. These multiple coats of cottoned materials, such as heavy clothes like kitenge and Khanga (a brightly coloured, thick and thin, rectangular cloth), are mostly used by the mothers to dress their newborn, especially those that are made up of cotton since they can retain warmth as compared to non-cotton materials. Some participants described that these materials were prepared

before delivery and are used to swaddle the baby from head to lower extremities to protect most of the areas that are sensitive to cold. The quote below from one of the participants attests this;

> *"First of all, you will cover the baby well with heavy clothes that do not permit the cold and which are not wet to free him from the cold" (P7-L)*

Another participant reported that, after the baby is born, it is very important to dress the baby in clothes that are made up of cotton as they do not permit cold, and the following quotes from the participants can justify this.

> *"Some clothes are made of materials that aren't able to maintain warmth, which can cause the baby to catch a cold once you cover him. But other clothes are made of cotton materials that can support warmth" (P5-H)*

Furthermore, participants also demonstrated that they cover newborns with many clothes to protect against diseases like pneumonia; however, this justifies their awareness of the complications related to hypothermia if left unattended. A P8-P delivered to her second baby attested.

> *"When the baby is born, I put him in socks, a hat, and a lot of wraps so that he doesn't get cold and become warm and prevent him from getting pneumonia" (P8-M)*

**ii. Preparing baby clothing before delivery**

Baby clothing refers to all baby materials that are used to protect the baby against hypothermia. These are widely used by many women who were interviewed, and most of the women demonstrated that before delivery, they make preparation, which involves buying, washing,

ironing, and packing them in a prepared bag ready to wait for the expected day for delivery. One of the participants from the interview explains this.

> *"Before I came to give birth, I made preparations for baby clothes like caps and socks; I bought, washed, and ironed them. This is because we were also taught during the clinic by nurses to prepare them to protect babies from colds after birth" (P1-P)*

However, it was noted that during the interview, some of the postnatal mothers described that they made preparations for their babies' clothing before delivery, and some didn't prepare them until they delivered, with the reason that they didn't prepare because they were not sure if they would deliver a live baby. One of the participants reported this

> "*These baby clothes were prepared by others; I did not prepare them. What happens if you make plans but end up not delivering the baby alive? Who is going to dress them?" (P4-M)*

**Theme two: Diversity of room temperature controlling measures**

During an interview, the participants highlighted that to keep the baby warm, there is a need to control the external environment and keep the room warm including closing the window and doors, switching off the fan if available, using of charcoal stove to heat the room and switching on the electric bulb in the room throughout the night with the hopes, these measures can keep the room warm and finally will prevent the baby from catching cold hence preventing hypothermia. From this theme, three sub-themes were developed.

**i. Maintaining indoor heating to warm the room**

Unwanted air current from entering the room is a key challenge that predisposes newborns to hypothermia. To prevent this, the majority of the postnatal mother explained that when they are at home, they close windows, and curtains and switch off the fan to maintain the warmth of the room. Furthermore, other participants use the charcoal stove to warm the room. This practice they do, especially during the winter period, by placing a charcoal stove far away from the bed, either near the door or in the living room, and the heat that will be generated will spread to all rooms and finally to the place where the baby is sleeping. Despite being aware of the consequences of using the charcoal stove in the room, the participants who were interviewed still use it as a preventive practice for newborn hypothermia. As one mother reported:

> "*As for the house, it depends on the environment where you live. Maybe the house has big windows that let in the cold. You need to make a lot of effort to keep the curtains closed to make sure that any part does not let in the cold*" *(P4-M)*

Another participant said that:

> *"When I return home, I have to close the windows and doors, and I have to switch off the fan while at home" (P3-P)*

Another mother said

> *"Right now, I am a parent, and the room I am going to use should be warm all the time that heat helps the baby, and me too……… I used to light the charcoal stove to boil the soup and water, so the heat would spread in the room. In many cases, the charcoal stove is also harmful,; for that reason, I always put it in the living room, and its heat will spread throughout the house, so the child gets air and heat too, because let's not just say heat, even air has its benefits" (P10-P)*

### ii. Maintaining indoor lighting to increase room temperature

Indoor lighting plays a great role in maintaining the room temperature through heat generation as adequate lighting contributes to an indoor temperature which helps to warm the room and prevent the occurrence of hypothermia among newborns. One of the participants reported that they use electric bulbs or solar bulbs that they switch on throughout the night to maintain the room temperature. This practice prevents newborn hypothermia.

The participant stated that:

> *"Also, switching on an electric light bulb throughout the night, even if you use solar light, helps to increase the temperature in the room" (P7-L)*

Another participant reported that:

> *"In the room, I will make sure to close the windows, the room should not have a fan, and the electric light should be left on so that it can help generate heat, when the baby sleeps, the lights should be left on, like electric bulbs at night" (P12-L)*

**Theme three: Skin-to-skin and feeding practices**

The interview revealed that postnatal mothers use skin-to-skin and feeding practices as a preventive measure against newborn hypothermia. Other participants mentioned that after the baby was delivered, the baby was placed on their chests by the health care providers, and also asked to start breastfeeding immediately as a way of creating a bond with their babies as well as protecting them from getting hypoglycemia, which will lead to hypothermia. Skin to skin is not commonly practised among mothers since few participants mentioned it as a way of preventing newborn hypothermia some of the participants further mentioned that after delivery, they don't practice skin-to-skin contact at their practices because the cord stump is still wet and intact; maybe if the

cord drops off also, they said skin-to-skin is for low-birth-weight babies and not for the full term, and also the initiation of breastfeeding was determined by the condition of the baby, such as crying, sleep, mothers' activities after delivery, and early handling of the baby to them from health care providers. Hence, skin-to-skin contact and feeding practices enable the baby to get heat through either flow of the mother's heat to the baby through skin-to-skin contact and also through the milk that breastfeeding contains a lot of calories, which also is the source of heat. The three sub-themes were identified under this theme.

**i. Keep a baby in physical contact with the mother for warmth**

The narratives show that physical contact, like putting the baby on the mother's chest, is non-existent to almost all of the participants, with a few mothers who were able to mention and practice, especially after delivery. Most of this physical closeness is done after the second stage of labour, when the baby is delivered, the baby is placed on the mother's chest by the health care professional to gain warmth from the mother, but not a regular practice for these post-natal mothers. Furthermore, other mothers demonstrate that putting the baby under the mother's chest is for low-birth-weight babies and not for babies with normal birth weight.

The above statement was explained by the following participant

> *"I also know that when a baby is underweight, he is placed in a kangaroo to get the warmth of his/her mother, but I do not know how it increases the temperature, but I only know that it is for babies with low weight" (P1-P)*

Another participant explained that

*"Putting the baby on the chest, skin to skin was done after giving birth, the baby was placed after I did not put him again and I have never done that again even with other children" (P7-L)*

Furthermore, other mothers are afraid to have physical closeness with their babies because the stump is still fresh, and physical contact with the baby's abdomen will hurt the stump. This can be justified by the following statement from one of the participants

*At the moment, I can't because I see that the umbilical cord has not been dropped off. On my side, maybe what I can do is, when the cord is dropped off, it will be fine. Maybe I can hurt the umbilical cord of the baby. I think that is the main reason why you cannot put the baby on the chest because the umbilical cord has not dropped off (P12-L).*

**ii. Breastfeeding practices**

Also, breastfeeding practice was mentioned by the mother as a way of preventing newborn hypothermia among newborns. One mother said during feeding, the baby is kept near the mother's abdomen, hence there is a transfer of heat from the mother to the baby, which also helps to keep the baby warm.

*"Also, the breastfeeding method helps because I am warm here and my milk is also warm, so when I breastfeed the baby, the breast milk is going to reduce the cold" (P11-H)*

Another mother said

*The early breastfeeding method can help the child to warm up because when you breastfeed, you hug him, and hence, he gets his mother's warmth (P6-H)*

Moreover, mothers do not perceive breastfeeding as a measure that should be carried out in a rigid schedule and are therefore oriented on feeding on demand, such as crying of the babies, handling of the baby by health care providers and completion of activities such as bathing carried out by the mothers after delivery

> *"…….. What helps me know that he wants to suckle is that you can find him crying because of hunger. When you see him calm, you will know that it was hunger" (P11-H)*

**Theme four: Body care measures**

Body care measures involve bathing activities and topical oil application on the newborn's skin to enhance warmth and promote comfort and sleep. Participants during the interview explained that babies need to receive care after delivery, and one of the cares is the use of oil to keep the skin smooth and warm after wiping or bathing using warm water to remove dirt, to promote a good feeling for the baby, and to enable the baby to sleep well. These measures were described by post-natal mothers aimed at softening the skin, and promoting skin warmth, and are done a few hours after delivery. Within this theme, two sub-themes emerge: massaging the newborn with oil to maintain skin warmth and bathing practices.

**i. Massaging the newborn with oil to maintain skin warmth**

Applying oil to baby skin may be beneficial—, possibly through physical stimulation while applying the oil, which also improves thermo-regulation. Newborn's massage, used for preventive as well as therapeutic processes, participants reported using coconut or parachute oil to improve baby's skin and warmth. As one mother described that

> *"I apply coconut oil on the baby to soften her skin and the coconut oil also helps to keep the baby warm" (P9-P)*

Coconut or parachute oil massage is common and all post-natal mothers are reported to engage in this behaviour. Applying oil to the baby's skin after delivery is a common practice that was demonstrated by the mothers. Participants also highlighted the use of coconut oil as a means of warming the baby. Coconut or parachute oil was used to improve skin condition and to reduce the incidence of hypothermia by reducing trans-epidermal water loss, hence improving thermoregulation.

Another participant reported;

> *"There is also applying coconut oil to the baby; you massage the baby with oil, making the baby feel warm. It's coconut oil. I used to massage the neck, legs, and just the whole body, and when you bathe the baby, you have to massage with oil because when you put the baby in the water, he becomes cold……., so he warms quickly" (P8-M)*

### ii. Bathing practices

The practice of wiping or bathing newborns within health facilities after birth is prevalent due to cultural and knowledge differences, contradicting the standard care of newborn care. Wiping or bathing of the newborn even before 24 hours is common in our setting, since after delivery, the presence of vernix caseosa and blood on the newborn's skin is perceived as dirty. Therefore, the mothers described using lukewarm water obtained from home and soft clothes obtained from local shops to bathe their babies to promote comfort, smooth sleep, and warmth for the baby.

This statement can be justified by the following participants as reported:

*"I wiped the baby because I noticed that the blood hadn't been wiped off thoroughly. Maybe he wasn't wiped too much due to his skin being soft, but because he continues to be here, I wiped him again. To wipe the baby, I need a soft cloth and lukewarm water. We usually start to wipe him after a few hours post-delivery—not soon after delivery, but before the cold comes in" (P4-M).*

Another participant explained:

*"In my experience, I bathe the baby with warm water twice a day at noon and before sundown to make it comfortable and so that he can sleep well" (P6-H)*

## Discussion

The study explored individualised preventive practices for newborn hypothermia, in which 12 post-natal mothers participated, and revealed that different preventive practices and other practices were minimally practised.

Interviewed post-natal mothers agree on the importance of thermal care to the newborn from birth to 28 days. The research findings indicate that post-natal mothers take different measures to protect their newborn from getting hypothermia, some of the initiatives are body covering measuring, skin-to-skin and feeding practices, prebonding, diversity of room temperature controlling measures, preparing baby clothes before delivery and body care measures such as the use of swaddling materials like heavy cotton clothes, baby costumes like baby shawl, blankets capes and hat.

But is not apparent all mothers take these measures as required to protect their newborn against catching a cold, some of the preventive practices such as skin-to-skin contact are not well practised

and are avoided due to fear of hurting the stump as well as breastfeeding practices are not initiated timely and their initiation is determined by the state of their babies either crying, sleeping and awake. According to the West Guji Zone South Ethiopia study, a cross-sectional study by Wako (9) indicated that drying and wrapping, head covering and early initiation of breastfeeding were common practices while delayed bathing and skin-to-skin contact were infrequently practised(16). This is also similar to our findings which have indicated some of the preventive practices that are taken into consideration were head covering and wrapping in heavy clothes.

Another study done in South India indicated that rooming in and covering with old cotton clothes was among the preventive practices that were performed by the mothers to protect their newborn against hypothermia, while skin-to-skin care was not common practice, these findings were in alignment with our findings which also revealed room in practices, use of heavy cotton material to cover the babies were among of the findings that were taken into consideration by the postnatal mothers in the study area. Similarly, the findings were also consistent with the study done in Kenyata National Hospital, Kenya, a mixed study on thermoregulation practices among mothers with newborn babies, which indicated that rooming in and the use of warm clothes were among thermoregulation practices to protect their babies against hypothermia(17). Also, these findings are similar to the study conducted in Northern India and Malawi, which also found that community members were not familiar with skin-to-skin care and did not practice it (18,19).

Also, the study findings are similar to the study conducted in Ghana to assess the knowledge and quality of essential newborn care practices in La Dade Kotopon Municipality, which indicated that some practices used in keeping babies warm were keeping close to the mother's skin, wrapping with warm clothes, and bathing of the baby with warm water (20).

Our findings were contrasted with the study findings done in Islamabad capital territory in Pakistan that assessed essential newborn care practices in squatters' settlements and that of Kenya and revealed that skin-to-skin contact was practised to more than half of the mothers (57%) within ten minutes of birth(21,22), this indicates that skin to skin contact is an evidence-based intervention for regulation and maintenance of newborn body temperature and is also the catalyst for breastfeeding.

Therefore, this study provides a critical evidence gap in newborn hypothermia prevention practices specific to the Kilimanjaro region, which can be generalised to lower- and middle-income countries, revealing an implementation gap despite thermal care practices and policies established by the World Health Organisation.

**Strengths and limitations of the study**

The study provides first-hand preventive practices of newborn hypothermia among postnatal mothers. Moreover, Triangulation was done at the data source level. The study also has limitations, like the study didn't explore the recommended practices versus those not recommended. Also, further interviews were not conducted to ensure data adequacy after saturation was reached. The study relied on mothers' opinions and failed to account for the experiences of health professionals, such as midwives and paediatricians, and the study did not triangulate data from multiple sources, such as from nurses and midwives, which lead to selection bias.

**Conclusion**

In conclusion, body covering measures, diversity of room temperature controlling measures, skin-to-skin contact and feeding practice, and body care measures were among the preventive practices elucidated by post-natal mothers in the study area. However, some preventive practices, like

indoor heating using charcoal stoves to warm the room, could be avoided in order to improve newborn and maternal health. Furthermore, skin-to-skin contact, the commencement of breastfeeding within one hour, and covering newborns with caps and socks are priority practices for newborn hypothermia prevention, requiring suitably tailored behaviour modification interventions among post-natal mothers.

## Recommendations

### For further studies

1. The cause of the lack of skin-to-skin care and delayed initiation of breastfeeding should be further explored for a potential solution
2. Further studies, an observation study should be carried out to observe the real practices of post-natal mothers, like skin-to-skin contact, which will help to quantify these practices

### For action

1. The Ministry of Health should develop multifaceted prevention strategies for newborn hypothermia, which should include community-based intervention, empowering postnatal mothers to adhere to recommended practices, and improving the availability of thermal care equipment in the healthcare system.
2. Post-natal mothers should be trained in thermal care during antenatal care contacts to enhance the development of positive behaviours from conception to post-delivery on thermal care practices.

## Supporting file

S1 File: Interview guide

**Acknowledgement**

The research team appreciate the administration of the health facilities where this study was conducted.

**Funding source and conflict of interest**

The authors have no funding or conflicts of interest to disclose

**Author contributions**

**Conceptualization**: Emmanuel Daniel, Saada A. Seif, Walter C. Millanzi

**Methodology**: Emmanuel Daniel, Saada A. Seif, Walter C. Millanzi

**Investigation**: Emmanuel Daniel

**Data curation**: Emmanuel Daniel

**Formal analysis**: Emmanuel Daniel, Saada A. Seif, Walter C. Millanzi

**Supervision**: Saada A. Seif, Walter C. Millanzi

**Writing -original draft**: Emmanuel Daniel

**Writing -review & editing**: Emmanuel Daniel, Saada A. Seif, Walter C. Millanzi

**References**


1. World Health Organization. World Health Organization- Thermal protection of the newborn: practical guide. Maternal and Newborn Health/ Safe Motherhood Unit Division of Retroductive Health. 2013;1–68.

2. Sharkh AA, Ighneimat F, Khdour YF, Aladam NY. Factors contributing to neonatal mortality in a tertiary center in south West Bank : A single- center retrospective cohort study. PLOS Glob Public Heal 5(7) e0004885 [Internet]. 2025;1–9. Available from: https://doi.org/10.1371/journal.pgph.0004885

3. TDHS. Tanzania demographic and health survey [Internet]. TDHS. 2022. Available from: https://dhsprogram.com/pubs/pdf/FR382/FR382.pdf

4. Nations U. The Sustainable Development Goals Report 2019. United Nations Publ issued by Dep Econ Soc Aff [Internet]. 2019;64. Available from: https://unstats.un.org/sdgs/report/2022/%0Ahttps://www.un-ilibrary.org/content/books/9789210018098%0Ahttps://www.un-ilibrary.org/content/books/9789210478878

5. Shah I, Paudyal L, Soti H. Descriptive Cross-Sectional Study on Mother ’ S Knowledge. J Univers Coll Med Sci [Internet]. 2022;10(01):62–6. Available from: https://doi.org/10.3126/jucms.v10i01.47244

6. Srivastava A, Saxena S, Srivastava P, Mahmood SE, Pandey R, Saxena A. Newborn thermal care in western Uttar Pradesh — gap analysis between knowledge and practices. Arch Public Heal [Internet]. 2022;1–10. Available from: https://doi.org/10.1186/s13690-022-00809-2

7. Fenta Kebede B, Dagnaw Genie Y, Yetwale Hiwot A, Biyazin T, Abebe B. Early Newborn Bath Practice and Its Associated Factors in Jimma, South West Ethiopia, 2021. Pediatr Heal Med Ther. 2022;Volume 13:43–52.

8. Demtse AG, Pfister RE, Nigussie AK, McClure EM, Ferede YG, Tazu Bonger Z, et al. Hypothermia in Preterm Newborns: Impact on Survival. Glob Pediatr Heal [Internet]. 2020;7. Available from: https://doi.org/10.1177/2333794X20957655

9. Wako WG, Beyene BN, Wayessa ZJ, Fikrie A, Amaje E. Assessment of neonatal thermal cares: Practices and beliefs among rural women in West Guji Zone, South Ethiopia: A cross-sectional study. PLOS Glob Public Heal [Internet]. 2022;2(6):e0000568. Available from: http://dx.doi.org/10.1371/journal.pgph.0000568

10. Memon J, Holakouie-Naieni K, Majdzadeh R, Yekaninejad MS, Garmaroudi G, Raza O, et al. Knowledge, attitude, and practice among mothers about newborn care in Sindh, Pakistan. BMC Pregnancy Childbirth [Internet]. 2019;19(1):1–9. Available from: https://link.springer.com/article/10.1186/s12884-019-2479-0

11. Tulelo PM, Mulaudzi FM. Indigenous neonatal feeding and bathing practices of caregivers in Vhembe district, Limpopo Province. Heal SA Gesondheid [Internet]. 2021;26:1–8. Available from: https://pmc.ncbi.nlm.nih.gov/articles/PMC8661288/

12. Khan SM, Timothy E, Singh K, Carvajal- L. Thermal care of newborns : drying and bathing practices in Malawi and Bangladesh. J Glob Health [Internet]. 2018;8(1):1–10. Available from: https://pmc.ncbi.nlm.nih.gov/articles/PMC5943716/

13. Nowell LS, Norris JM, White DE, Moules NJ. Thematic Analysis: Striving to Meet the Trustworthiness Criteria. Int J Qual Methods. 2017;16(1):1–13.

14. Braun V, Clarke V. Using thematic analysis in psychology. Qual Res Psychol. 2006;3(2):77–101.

15. Brannen J. Mixing methods: The entry of qualitative and quantitative approaches into the research process. Int J Soc Res Methodol Theory Pract. 2005;8(3):173–84.

16. Sjömar J, Ottesen H, Banik G, Rahman AE, Thernström Blomqvist Y, Rahman SM, et al. Exploring caregivers' experiences of Kangaroo Mother Care in Bangladesh: A descriptive qualitative study. PLoS One [Internet]. 2023;18(1 January):1–16. Available from: https://journals.plos.org/plosone/article?id=10.1371/journal.pone.0280254

17. Wanjiru JW, Makworo D, Simba JM. Thermoregulation Practices among Mothers with New-Born Babies Attending Kenyatta National Hospital, Kenya. Galore Int J Heal Sci Res [Internet]. 2021;6(3):82–9.

18. Qazi M, Saqib N, Raina R. Knowledge regarding prevention of hypothermia in newborns among mothers in Northern India. Int J Res Med Sci [Internet]. 2019;7(5):1727. Available from: https://pesquisa.bvsalud.org/portal/resource/pt/sea-211466

19. Nyondo-Mipando AL, Kinshella MLW, Hiwa T, Salimu S, Banda M, Vidler M, et al. Mothers' quality of life delivering kangaroo mother care at Malawian hospitals: a qualitative study. Health Qual Life Outcomes [Internet]. 2021;19(1):1–8. Available from: https://doi.org/10.1186/s12955-021-01823-8

20. Ayete-Nyampong J, Udofia EA. Assessment of knowledge and quality of essential newborn care practices in La Dade Kotopon Municipality, Ghana. PLoS One [Internet]. 2020;15(8 August 2020):1–15. Available from: http://dx.doi.org/10.1371/journal.pone.0237820

21. RH H, SH H, TR R, KH H, JB B, FE E. Assessment of Essential Newborn Care Practices in the Squatter Settlements of Islamabad Capital Territory in Pakistan. J Pediatr Child Heal Care [Internet]. 2021;6(2). Available from: https://austinpublishinggroup.com/pediatrics-child-health-care/fulltext/jpchc-v6-id1043.pdf

22. Bhuiya NA, Liu S, Muyodi D, Bucher SL. Feasibility and acceptability of a novel biomedical device to prevent neonatal hypothermia and augment Kangaroo Mother Care in Kenya: Qualitative analysis of focus group discussions and key Informant Interviews. PLOS Glob Public Heal [Internet]. 2024;4(4):1–20. Available from: http://dx.doi.org/10.1371/journal.pgph.0001708

# Tittle: Exploring preventive practices of newborn hypothermia among post-natal mothers in selected public health facilities in Kilimanjaro region: Qualitative descriptive study

**Ref: Submission ID: PONE-D-25-62885**

**Journal requirement**

| S/N | JOURNAL REQUIREMENT | AUTHOR'S RESPONSE |
|---|---|---|
| 1 | When submitting your revision, we need you to address these additional requirements. 1. Please ensure that your manuscript meets PLOS ONE's style requirements, including those for file naming. The PLOS ONE style templates can be found at https://journals.plos.org/plosone/s/file?id=wjVg/PLOSOne_formatting_sample_main_body.pdf and https://journals.plos.org/plosone/s/file?id=ba62/PLOSOne_formatting_sample_title_authors_affiliations.pdf | Thank you. We have made sure that our manuscript to abide with PLOS ONE style requirement |
| 2 | Your ethics statement should only appear in the Methods section of your manuscript. If your ethics statement is written in any section besides the Methods, please delete it from any other section. | Thank you for the valuable comment. The ethical statement has been included in the method section |
| 3 | In the online submission form, you indicated that all data will be available from the corresponding author upon request. | Concerning the data set of the underlying results of the manuscript. All relevant data are within the manuscript and its supporting files. Raw data, such as transcripts and translations, cannot be shared publicly because of the confidentiality assurance given to participants at the time of consent. Hence, the data can be accessed through the institutional research review committee (IRRC) of the University of Dodoma through drpc@udom.ac.tz after being processed and accepted by the committee. |

| | | |
|---|---|---|
| 4 | Please amend your authorship list in your manuscript file to include author Emmanuel Daniel. | Author Emmanuel Daniel is included in the revised manuscript |
| 5 | Please amend the manuscript submission data (via Edit Submission) to include author Emmanuel Daniel Kija. | Author Emmanuel Daniel Kija is omitted in the submission and in manuscript |
| 6 | . Please remove all personal information, ensure that the data shared are in accordance with participant consent, and re upload a fully anonymized data set. Note: spreadsheet columns with personal | All person information is removed in the revised manuscript |
| 7 | Please include captions for your Supporting Information files at the end of your manuscript, and update any in-text citations to match accordingly. Please see our Supporting Inf | Thank you, caption for supporting information file is included in text citation |

**REVIEWER #1: AUTHOR'S RESPONSES TO COMMENTS**

| S/N | REVIEWER'S COMMENT | AUTHOR'S RESPONSE | PG. NUMBER |
|---|---|---|---|
| 1 | Why were the demographic information of the two participants interviewed to ensure data adequacy not reported? And were the interviews transcribed and analyzed? Please explain in detail and if not included in the analysis, amend this section of the results and explicitly state in the limitations section that further interviews were not conducted to ensure data adequacy after saturation was reached | Thank you for your constructive comments. Amendments have been made to comply with your comment | Page 10, 26 and 27 |
| 2 | Add a column in Table 2 and report the open codes for each subtheme to facilitate tracking of findings and provide comprehensive findings for future researchers. | Column in table 2 has been added to include open codes from participants quotes | Page 13 -15 |
| 3 | What is described in the text on line 403 for the number of subthemes in theme " " does not match the number of subthemes in the rest of the text and in Table 2. Please review and correct. | Thank you for the constructive comment. The comment has been addressed in the revised manuscript | Pages 21 |
| 4 | Undoubtedly, the limitations of the study, especially methodological ones, include more issues that researchers need to pay attention to and carefully point out, including the use of only mothers' opinions and the failure to address the experiences of health professionals such as midwives and pediatricians who interacted with these mothers and infants and who have shared experiences. Meanwhile, triangulation was also created at the data source level, adding to the strength of the study | The limitations of the study have been revised and now include the suggestion that the study relied on mothers' opinions and failed to account for the experiences of health professionals, such as midwives and paediatricians. | Pages 26 and 27 |

**REVIEWER #2: AUTHOR'S RESPONSES TO COMMENTS**

| S/N | REVIEWER’S COMMENT | AUTHOR’S RESPONSE | PG. NUMBER |
|---|---|---|---|
| 1 | Please make the manuscript region-specific. There is a difference in the care provided in the high-, middle- and low-income countries.<br>• Add these parameters in the introduction, discussion and methods as well. | Thank you for the constructive comment. The study explored more about lower-middle-income countries | All manuscript |
| 2 | There is need to make the description of themes in a crisper and shortened format.<br>• The quotes of the patients may also be tabulated along with the themes | Thanks for the comment, themes remained as it is short according to our view and open codes instead of patient quotes have been added in Table 2 to track findings, since patient codes may be tracked in the results | Page 13-15 |
| | . Our settings should be changed to a hospital setting.<br>• Remove word semi semi-structured duplication | Setting has been included in the revised manuscript, and the word semi has been omitted | Page 2 |
| | Temperature fluctuation does not lead to asphyxia, and has to be deleted | Changes have been made in the revised manuscript | Page 3 |
| | This also shows there was a need to intervene appropriately by providing appropriate thermal care. This would be one of the ways to reduce NMR, not the only one. | Thank you for the comment, the sentence has been revised and refined | Page 3 |
| | What is the meaning of double increase rather than decline | The meaning of double increase rather than decline means that the newborn mortality is still high compared to the Sustainable Development Goal of reducing newborn mortality to 12 per 1000 live births by 20230, as the newborn mortality rate in Tanzania is 24 per 1000 live births. | Page 3 |

| | | | |
|---|---|---|---|
| | Ensure uniform citation formatting according to journal requirements | All citations have been formatted in the revised manuscript | All pages in a manuscript |
| | Strengthen the "knowledge gap" statement: explicitly indicate what is missing in existing literature from Tanzania/Kilimanjaro. | Strengthening the "knowledge gap" statement is done in the revised manuscript | Page 4 |
| | The rationale of the study being carried out in that particular region needs to be tailored according to the region and so needs modification in the wording | Modification has been carried out to make it clear | Page 4 |
| | The rationale given for the usage of this design may be avoided as it's a known fact. | The comment has been incorporated in the revised manuscript | Page 5 |
| | Researchers chose the purposeful sampling technique to identify the population likely to possess detailed information on newborn hypothermia preventive practices" introduces a significant bias in the study. The mothers who know how to prevent in a detailed manner will be able to provide biased information. | We appreciate your constructive feedback and had added limitation paragraph in the revised manuscript to clearly address this comment | Page 26 and 27 |
| | Majority of the women were entrepreneur, employed. This also leads to bias. Describe strategies used to ensure maximum variation in sampling. | Thank you for this valid concern about potential selection bias due to the overrepresentation of entrepreneurs and employed women. We incorporated strategies for maximum variation in our study to capture diverse socioeconomic and occupational perspectives through stratification of participants by contextual and key demographic characteristics to ensure heterogeneity beyond employment status, such as parity, education level, facility level, and experiences | Page 6 and 12 |

| | | | |
|---|---|---|---|
| | Mention if the interview guide was pilot-tested or iteratively modified. | Thank you for the valid comment, the tool was piloted and details has been included in revised manuscript | Page 8 |
| | Add contextual details of the facilities(rural/urban, patient flow, type of maternity services). • Explain why these specific facilities were chosen | Thanks for the comment, contextual details of the facilities has been added in the revised manuscript | Page 6 |
| | Describe how coding disagreements were resolved. | Coding disagreements were reviewed and discussed by the research team as explained in the revised manuscripts | Page 10 |
| | Mention number of participants contributing to each theme (COREQ requirement). | Thank you for the constructive comment. An explanation about the number of participants contributing to each theme is added in the revised manuscript | Page 9 |
| | Add a short subsection on the study's strengths (currently absent). | Subsection of strength has been added in the current manuscripts | Page 26 |
| | Provide a clearer articulation of how this work adds to regional evidence. | Articulation of how this works adds to regional evidence has been added in the revised manuscript | Page 26 |
| | References need rewriting. | Thank you for the constructive feedback, all references have been formatted again in the revised manuscript | Page 29,30,31 and 32 |
