# Supplementary material for: Exploring preventive practices of newborn hypothermia among post-natal mothers in selected public health facilities in Kilimanjaro region: Qualitative descriptive study
Source: PLoS One. 2026 Sep 10;21(9):e0357756. doi: 10.1371/journal.pone.0357756 (PMC13561377; doi:10.1371/journal.pone.0357756)
Supplement: Supplementary file 4 [file pone.0357756.s004.docx]

**Tittle: Exploring preventive practices of newborn hypothermia among post-natal mothers in selected public health facilities in Kilimanjaro region: Qualitative descriptive study**

**Ref: Submission ID: PONE-D-25-62885**

**Journal requirement**

| **S/N** | **JOURNAL REQUIREMENT** | **AUTHOR’S RESPONSE** |
| --- | --- | --- |
| 1 | When submitting your revision, we need you to address these additional requirements. 1. Please ensure that your manuscript meets PLOS ONE's style requirements, including those for file naming. The PLOS ONE style templates can be found at https://journals.plos.org/plosone/s/file?id=wjVg/PLOSOne_formatting_sample_main_body.pdf and https://journals.plos.org/plosone/s/file?id=ba62/PLOSOne_formatting_sample_title_authors_affiliations.pdf | Thank you. We have made sure that our manuscript to abide with PLOS ONE style requirement |
| 2 | Your ethics statement should only appear in the Methods section of your manuscript. If your ethics statement is written in any section besides the Methods, please delete it from any other section. | Thank you for the valuable comment. The ethical statement has been included in the method section |
| 3 | In the online submission form, you indicated that all data will be available from the corresponding author upon request. | Concerning the data set of the underlying results of the manuscript. All relevant data are within the manuscript and its supporting files. Raw data, such as transcripts and translations, cannot be shared publicly because of the confidentiality assurance given to participants at the time of consent. Hence, the data can be accessed through the institutional research review committee (IRRC) of the University of Dodoma through [drpc@udom.ac.tz](mailto:drpc@udom.ac.tz) after being processed and accepted by the committee. |
| 4 | Please amend your authorship list in your manuscript file to include author Emmanuel Daniel. | Author Emmanuel Daniel is included in the revised manuscript |
| 5 | Please amend the manuscript submission data (via Edit Submission) to include author Emmanuel Daniel Kija. | Author Emmanuel Daniel Kija is omitted in the submission and in manuscript |
| 6 | . Please remove all personal information, ensure that the data shared are in accordance with participant consent, and re upload a fully anonymized data set. Note: spreadsheet columns with personal | All person information is removed in the revised manuscript |
| 7 | Please include captions for your Supporting Information files at the end of your manuscript, and update any in-text citations to match accordingly. Please see our Supporting Inf | Thank you, caption for supporting information file is included in text citation |

**REVIEWER #1: AUTHOR'S RESPONSES TO COMMENTS**

| **S/N** | **REVIEWER’S COMMENT** | **AUTHOR’S RESPONSE** | **PG. NUMBER** |
| --- | --- | --- | --- |
| 1 | Why were the demographic information of the two participants interviewed to ensure data adequacy not reported? And were the interviews transcribed and analyzed? Please explain in detail and if not included in the analysis, amend this section of the results and explicitly state in the limitations section that further interviews were not conducted to ensure data adequacy after saturation was reached | Thank you for your constructive comments. Amendments have been made to comply with your comment | Page 10, 26 and 27 |
| 2 | Add a column in Table 2 and report the open codes for each subtheme to facilitate tracking of findings and provide comprehensive findings for future researchers. | Column in table 2 has been added to include open codes from participants quotes | Page 13 -15 |
| 3 | What is described in the text on line 403 for the number of subthemes in theme " " does not match the number of subthemes in the rest of the text and in Table 2. Please review and correct. | Thank you for the constructive comment. The comment has been addressed in the revised manuscript | Pages 21 |
| 4 | Undoubtedly, the limitations of the study, especially methodological ones, include more issues that researchers need to pay attention to and carefully point out, including the use of only mothers' opinions and the failure to address the experiences of health professionals such as midwives and pediatricians who interacted with these mothers and infants and who have shared experiences. Meanwhile, triangulation was also created at the data source level, adding to the strength of the study | The limitations of the study have been revised and now include the suggestion that the study relied on mothers' opinions and failed to account for the experiences of health professionals, such as midwives and paediatricians. | Pages 26 and 27 |

**REVIEWER #2: AUTHOR'S RESPONSES TO COMMENTS**

| S/N | REVIEWER’S COMMENT | AUTHOR’S RESPONSE | PG. NUMBER |
| --- | --- | --- | --- |
| 1 | Please make the manuscript region-specific. There is a difference in the care provided in the high-, middle- and low-income countries.  • Add these parameters in the introduction, discussion and methods as well. | Thank you for the constructive comment. The study explored more about lower-middle-income countries | All manuscript |
| 2 | There is need to make the description of themes in a crisper and shortened format. • The quotes of the patients may also be tabulated along with the themes | Thanks for the comment, themes remained as it is short according to our view and open codes instead of patient quotes have been added in Table 2 to track findings, since patient codes may be tracked in the results | Page 13-15 |
|  | . Our settings should be changed to a hospital setting. • Remove word semi semi-structured duplication | Setting has been included in the revised manuscript, and the word semi has been omitted | Page 2 |
|  | Temperature fluctuation does not lead to asphyxia, and has to be deleted | Changes have been made in the revised manuscript | Page 3 |
|  | This also shows there was a need to intervene appropriately by providing appropriate thermal care. This would be one of the ways to reduce NMR, not the only one. | Thank you for the comment, the sentence has been revised and refined | Page 3 |
|  | What is the meaning of double increase rather than decline | The meaning of double increase rather than decline means that the newborn mortality is still high compared to the Sustainable Development Goal of reducing newborn mortality to 12 per 1000 live births by 20230, as the newborn mortality rate in Tanzania is 24 per 1000 live births. | Page 3 |
|  | Ensure uniform citation formatting according to journal requirements | All citations have been formatted in the revised manuscript | All pages in a manuscript |
|  | Strengthen the “knowledge gap” statement: explicitly indicate what is missing in existing literature from Tanzania/Kilimanjaro. | Strengthening the “knowledge gap” statement is done in the revised manuscript | Page 4 |
|  | The rationale of the study being carried out in that particular region needs to be tailored according to the region and so needs modification in the wording | Modification has been carried out to make it clear | Page 4 |
|  | The rationale given for the usage of this design may be avoided as it’s a known fact. | The comment has been incorporated in the revised manuscript | Page 5 |
|  | Researchers chose the purposeful sampling technique to identify the population likely to possess detailed information on newborn hypothermia preventive practices” introduces a significant bias in the study. The mothers who know how to prevent in a detailed manner will be able to provide biased information. | We appreciate your constructive feedback and had added limitation paragraph in the revised manuscript to clearly address this comment | Page 26 and 27 |
|  | Majority of the women were entrepreneur, employed. This also leads to bias. Describe strategies used to ensure maximum variation in sampling. | Thank you for this valid concern about potential selection bias due to the overrepresentation of entrepreneurs and employed women. We incorporated strategies for maximum variation in our study to capture diverse socioeconomic and occupational perspectives through stratification of participants by contextual and key demographic characteristics to ensure heterogeneity beyond employment status, such as parity, education level, facility level, and experiences | Page 6 and 12 |
|  | Mention if the interview guide was pilot-tested or iteratively modified. | Thank you for the valid comment, the tool was piloted and details has been included in revised manuscript | Page 8 |
|  | Add contextual details of the facilities(rural/urban, patient flow, type of maternity services). • Explain why these specific facilities were chosen | Thanks for the comment, contextual details of the facilities has been added in the revised manuscript | Page 6 |
|  | Describe how coding disagreements were resolved. | Coding disagreements were reviewed and discussed by the research team as explained in the revised manuscripts | Page 10 |
|  | Mention number of participants contributing to each theme (COREQ requirement). | Thank you for the constructive comment. An explanation about the number of participants contributing to each theme is added in the revised manuscript | Page 9 |
|  | Add a short subsection on the study’s strengths (currently absent). | Subsection of strength has been added in the current manuscripts | Page 26 |
|  | Provide a clearer articulation of how this work adds to regional evidence. | Articulation of how this works adds to regional evidence has been added in the revised manuscript | Page 26 |
|  | References need rewriting. | Thank you for the constructive feedback, all references have been formatted again in the revised manuscript | Page 29,30,31 and 32 |
