# Supplementary material for: Exploring preventive practices of newborn hypothermia among post-natal mothers in selected public health facilities in Kilimanjaro region: Qualitative descriptive study
Source: PLoS One. 2026 Sep 10;21(9):e0357756. doi: 10.1371/journal.pone.0357756 (PMC13561377; doi:10.1371/journal.pone.0357756)
Supplement: Supplementary file 5 [file pone.0357756.s006.docx]

**Tittle: Exploring preventive practices of newborn hypothermia among post-natal mothers in selected public health facilities in Kilimanjaro region: Qualitative descriptive study**

**Ref: Submission ID: PONE-D-25-62885**

**REVIEWER #1: AUTHOR'S RESPONSES TO COMMENTS**

| **S/N** | **REVIEWER’S COMMENT** | **AUTHOR’S RESPONSE** | **PG. NUMBER** |
| --- | --- | --- | --- |
| 1 | The number of participants in the method should be added and removed from the method | Thank you for constructive suggestion, number of participants has been included in the method section | Page 2 |
| 2 | The conclusion is not based on the findings. On what basis did you identify the need for education for mothers? It is suggested that it be revised. | Thanks for the comment, conclusion has been improved based on findings in the revised manuscript | Page 3 |
| 3 | The number of participants should be reported on the method section | Thank you for the comments, participants have been added in the method section in the revised manuscript | Page 7 |

**REVIEWER #2: AUTHOR'S RESPONSES TO COMMENTS**

| S/N | REVIEWER’S COMMENT | AUTHOR’S RESPONSE | PG. NUMBER |
| --- | --- | --- | --- |
| 1 | The English language may be made simpler for the wider audience. Esoteric words may be decreased. It will help the people in LMIC to understand and disseminate it better. | Thank you for the constructive comments, English used is simple and understandable even in LMIC | All manuscript |
|  |  |  |  |
