# Supplementary material for: Exploring preventive practices of newborn hypothermia among post-natal mothers in selected public health facilities in Kilimanjaro region: Qualitative descriptive study
Source: PLoS One. 2026 Sep 10;21(9):e0357756. doi: 10.1371/journal.pone.0357756 (PMC13561377; doi:10.1371/journal.pone.0357756)
Supplement: Supplementary file 6 [file pone.0357756.s007.docx]

**Tittle: Exploring preventive practices of newborn hypothermia among post-natal mothers in selected public health facilities in Kilimanjaro region: Qualitative descriptive study**

**Ref: Submission ID: PONE-D-25-62885**

**REVIEWER: AUTHOR'S RESPONSES TO COMMENTS**

| **S/N** | **REVIEWER’S COMMENT** | **AUTHOR’S RESPONSE** | **PG. NUMBER** |
| --- | --- | --- | --- |
| 1. | Check grammar: Line 48-49 These findings call for comprehensive 49 promoting a positive behaviour on thermal newborn protection. | Thank you for the constructive comment, we have worked to correct grammar in the revised manuscript | Page 2-3 |
| 2. | Improper capilization in line 57: Moderate hypothermia | Thank you, Moderate hypothermia has been corrected to moderate hypothermia in the revised manuscript | Page 3-line 54 |
| 3. | Make it simple sentence line 60-63 | Thanks, stipulated sentence has been made simple | Page 3 |
| 4. | Please clarify the correct phrase: epistemological position or ability | Thank you, the study approach, and design was reviewed again and written well in the revised manuscript | Page 5 |
| 5. | 272. table. Check spelling of ‘bag’ | Thanks, spelling for bag was checked and corrected | Page 14 |
| 6. | Line 344: Please check the spelling of “soup” | Thank you for the constructive comment, the spelling of soup is correct which mean that soup is a liquid food especially with meat, fish or vegetable stock as a base and often containing pieces of solid food- according to medical dictionary | Page 18 |
| 7. | The conclusion is not fully aligned with the study methodology. | Thank you for the comment, we have corrected our conclusion to align with our study methodology as suggested | Page 3 |
| 8. | Please consider whether concepts such as phenomenological integrity, prolonged engagement, intercoder reliability, and transferability are appropriate for this study design. If these concepts are retained, their use should be clearly justified and aligned with the stated qualitative methodology. Otherwise, they should be replaced with terminology that is consistent with a qualitative descriptive approach. | Thank you, concepts such as phenomenological integrity, prolonged engagement, intercoder reliability, and transferability have been revised to its applicability and the method section has been revised again to align with qualitative descriptive approach | Page 5-11 |
| 9. | Descriptions of the audio recorder's technical features are unnecessarily detailed and should be replaced with information that is methodologically relevant. | Thanks, your suggestion has been put into consideration. Description of audio recorders were removed in the revised manuscript | Page 8 |
| 10. | Several procedures also require further clarification, including the forward–backward translation process, participant recruitment, transcript verification (member checking), assessment of data saturation. | Thank you for the comment, a thorough clarification of forward backward translation process, participants recruitment, member checking and assessment of data saturation has been made for easy understanding, | Page 7,9 and 11 |
