# Supplementary material for: Exploring preventive practices of newborn hypothermia among post-natal mothers in selected public health facilities in Kilimanjaro region: Qualitative descriptive study
Source: PLoS One. 2026 Sep 10;21(9):e0357756. doi: 10.1371/journal.pone.0357756 (PMC13561377; doi:10.1371/journal.pone.0357756)
Supplement: Peer review history

# Peer review: Decision Letter 0

**DOI:** 10.1371/journal.pone.0357756.r001

**Authors:** Syeda Humaida Hasan

24 Feb 2026

Dear Dr. Daniel,

Thank you for submitting your manuscript to PLOS ONE. After careful consideration, we feel that it has merit but does not fully meet PLOS ONE’s publication criteria as it currently stands. Therefore, we invite you to submit a revised version of the manuscript that addresses the points raised during the review process.

Please submit your revised manuscript by Apr 10 2026 11:59PM. If you will need more time than this to complete your revisions, please reply to this message or contact the journal office at plosone@plos.org. When you're ready to submit your revision, log on to https://www.editorialmanager.com/pone/ and select the 'Submissions Needing Revision' folder to locate your manuscript file.

- A letter that responds to each point raised by the academic editor and reviewer(s). You should upload this letter as a separate file labeled 'Response to Reviewers'.
- A marked-up copy of your manuscript that highlights changes made to the original version. You should upload this as a separate file labeled 'Revised Manuscript with Track Changes'.
- An unmarked version of your revised paper without tracked changes. You should upload this as a separate file labeled 'Manuscript'.

If you would like to make changes to your financial disclosure, please include your updated statement in your cover letter. Guidelines for resubmitting your figure files are available below the reviewer comments at the end of this letter.

If applicable, we recommend that you deposit your laboratory protocols in protocols.io to enhance the reproducibility of your results. Protocols.io assigns your protocol its own identifier (DOI) so that it can be cited independently in the future. For instructions see: https://journals.plos.org/plosone/s/submission-guidelines#loc-laboratory-protocols. Additionally, PLOS ONE offers an option for publishing peer-reviewed Lab Protocol articles, which describe protocols hosted on protocols.io. Read more information on sharing protocols at https://plos.org/protocols?utm_medium=editorial-email&utm_source=authorletters&utm_campaign=protocols.

We look forward to receiving your revised manuscript.

Kind regards,

Syeda Humaida Hasan, Diploma in Child Health, FCPS (Pediatrics)

Academic Editor

PLOS One

Journal Requirements:

When submitting your revision, we need you to address these additional requirements.

1. Please ensure that your manuscript meets PLOS ONE's style requirements, including those for file naming. The PLOS ONE style templates can be found at

https://journals.plos.org/plosone/s/file?id=wjVg/PLOSOne_formatting_sample_main_body.pdf and https://journals.plos.org/plosone/s/file?id=ba62/PLOSOne_formatting_sample_title_authors_affiliations.pdf

2. Your ethics statement should only appear in the Methods section of your manuscript. If your ethics statement is written in any section besides the Methods, please delete it from any other section.

3. In the online submission form, you indicated that All data will be available from corresponding author upon request.

All PLOS journals now require all data underlying the findings described in their manuscript to be freely available to other researchers, either 1. In a public repository, 2. Within the manuscript itself, or 3. Uploaded as supplementary information.

This policy applies to all data except where public deposition would breach compliance with the protocol approved by your research ethics board. If your data cannot be made publicly available for ethical or legal reasons (e.g., public availability would compromise patient privacy), please explain your reasons on resubmission and your exemption request will be escalated for approval.

4. Please amend your authorship list in your manuscript file to include author Emmanuel Daniel.

5. Please amend the manuscript submission data (via Edit Submission) to include author Emmanuel Daniel Kija.

6. Please remove all personal information, ensure that the data shared are in accordance with participant consent, and re-upload a fully anonymized data set.

Note: spreadsheet columns with personal information must be removed and not hidden as all hidden columns will appear in the published file.

Additional guidance on preparing raw data for publication can be found in our Data Policy (https://journals.plos.org/plosone/s/data-availability#loc-human-research-participant-data-and-other-sensitive-data) and in the following article: http://www.bmj.com/content/340/bmj.c181.long.

7. Please include captions for your Supporting Information files at the end of your manuscript, and update any in-text citations to match accordingly. Please see our Supporting Information guidelines for more information: http://journals.plos.org/plosone/s/supporting-information.

8. If the reviewer comments include a recommendation to cite specific previously published works, please review and evaluate these publications to determine whether they are relevant and should be cited. There is no requirement to cite these works unless the editor has indicated otherwise.

[Note: HTML markup is below. Please do not edit.]

Reviewers' comments:

Reviewer's Responses to Questions

**Comments to the Author**

1. Is the manuscript technically sound, and do the data support the conclusions?

Reviewer #1: Partly

Reviewer #2: Partly

**********

2. Has the statistical analysis been performed appropriately and rigorously?

Reviewer #1: N/A

Reviewer #2: Yes

**********

3. Have the authors made all data underlying the findings in their manuscript fully available?

The PLOS Data policy

Reviewer #1: No

Reviewer #2: No

**********

4. Is the manuscript presented in an intelligible fashion and written in standard English?

Reviewer #1: No

Reviewer #2: Yes

**********

Reviewer #1: Dear Author,

Why were the demographic information of the two participants interviewed to ensure data adequacy not reported? And were the interviews transcribed and analyzed? Please explain in detail and if not included in the analysis, amend this section of the results and explicitly state in the limitations section that further interviews were not conducted to ensure data adequacy after saturation was reached.

Add a column in Table 2 and report the open codes for each subtheme to facilitate tracking of findings and provide comprehensive findings for future researchers.

What is described in the text on line 403 for the number of subthemes in theme " " does not match the number of subthemes in the rest of the text and in Table 2. Please review and correct.

Undoubtedly, the limitations of the study, especially methodological ones, include more issues that researchers need to pay attention to and carefully point out, including the use of only mothers' opinions and the failure to address the experiences of health professionals such as midwives and pediatricians who interacted with these mothers and infants and who have shared experiences. Meanwhile, triangulation was also created at the data source level, adding to the strength of the study.

Reviewer #2: You have beautifully described the in-depth interviews of 12 participants about the preventive practices in post-natal hospital wards. My comments and suggestions are as follows:

• Please make the manuscript region specific. There is difference in the care provided in the high-, middle- and low-income countries.

• Add these parameters in the introduction, discussion and methods as well.

• There is need to make the description of themes in a crisper and shortened format.

• The quotes of the patients may also be tabulated along with the themes.

• All questions and probes of the Interview guide are not addressed in the thematic analysis.

MY specific comments are as follows:

Abstract:

• Our settings should be changed to hospital setting.

• Remove word semi semi-structured duplication

Introduction

• Contert 0 to degree symbol.

• Temperature fluctuation does not lead to asphyxia, has to be deleted

• This also shows there was a need to intervene appropriately by providing appropriate thermal care. This would be one of the aspect of decreasing NMR, not the only one.

• The inference about the mother’s knowledge is not universal. As stated in this draft, this looks like an over assumption. The sentence Should be made contextual to very LMIC.

• Similarly line 73-75 have to be made region specific.

* What is the meaning of double increase rather than decline.

• Strengthen the “knowledge gap” statement: explicitly indicate what is missing in existing literature from Tanzania/Kilimanjaro.

• Ensure uniform citation formatting according to journal requirements.

• The rationale of the study being carried out in that particular region needs to be tailored according to the region and so needs modification in the wording.

Material and methods

• The rationale given for the usage of this design may be avoided as it’s a known fact.

• Sampling technique of “Researchers chose the purposeful sampling technique to identify the population likely to possess detailed information on newborn hypothermia preventive practices” introduces a significant bias in the study. The mothers who know how to prevent in a detailed manner will be able to provide biased information.

• Majority of the women were entrepreneur, employed. This also leads to bias. Describe strategies used to ensure maximum variation in sampling.

• Mention if the interview guide was pilot-tested or iteratively modified.

• Add contextual details of the facilities(rural/urban, patient flow, type of maternity services).

• Explain why these specific facilities were chosen.

• Describe how coding disagreements were resolved.

• Mention number of participants contributing to each theme (COREQ requirement).

Discussion

• Add a short subsection on the study’s strengths (currently absent).

• Provide a clearer articulation of how this work adds to regional evidence.

References

• References need rewriting. You should use some referemnce manager for smooth incorporation.

• Ref no 4 is incomplete

• Ref No 7 is not contextual and is incomplete Demtse AG, Pfister RE, Nigussie AK, et al. Hypothermia in Preterm Newborns: Impact on Survival. Glob Pediatr Health. 2020;7:2333794X20957655. Published 2020 Sep 13.

• Ref no 12, the journal name is missing.

• etc

**********

what does this mean?). If published, this will include your full peer review and any attached files.

If you choose “no”, your identity will remain anonymous but your review may still be made public.

**Do you want your identity to be public for this peer review?**  For information about this choice, including consent withdrawal, please see our Privacy Policy

Reviewer #1: No

Reviewer #2: No

**********

[NOTE: If reviewer comments were submitted as an attachment file, they will be attached to this email and accessible via the submission site. Please log into your account, locate the manuscript record, and check for the action link "View Attachments". If this link does not appear, there are no attachment files.]

To ensure your figures meet our technical requirements, please review our figure guidelines: https://journals.plos.org/plosone/s/figures

You may also use PLOS’s free figure tool, NAAS, to help you prepare publication quality figures: https://journals.plos.org/plosone/s/figures#loc-tools-for-figure-preparation.

NAAS will assess whether your figures meet our technical requirements by comparing each figure against our figure specifications.

# Author comment: Author response to Decision Letter 1

**DOI:** 10.1371/journal.pone.0357756.r002

30 Apr 2026

Tittle: Exploring preventive practices of newborn hypothermia among post-natal mothers in selected public health facilities in Kilimanjaro region: Qualitative descriptive study

Ref: Submission ID: PONE-D-25-62885

JOURNAL REQUIREMENT

1 When submitting your revision, we need you to address these additional requirements. 1. Please ensure that your manuscript meets PLOS ONE's style requirements, including those for file naming. The PLOS ONE style templates can be found at https://journals.plos.org/plosone/s/file?id=wjVg/PLOSOne_formatting_sample_main_body.pdf and https://journals.plos.org/plosone/s/file?id=ba62/PLOSOne_formatting_sample_title_authors_affiliations.pdf

Response: Thank you. We have made sure that our manuscript to abide with PLOS ONE style requirement

2 Your ethics statement should only appear in the Methods section of your manuscript. If your ethics statement is written in any section besides the Methods, please delete it from any other section.

Response: Thank you for the valuable comment. The ethical statement has been included in the method section

3 In the online submission form, you indicated that all data will be available from the corresponding author upon request.

Response: Concerning the data set of the underlying results of the manuscript. All relevant data are within the manuscript and its supporting files. Raw data, such as transcripts and translations, cannot be shared publicly because of the confidentiality assurance given to participants at the time of consent. Hence, the data can be accessed through the institutional research review committee (IRRC) of the University of Dodoma through drpc@udom.ac.tz after being processed and accepted by the committee.

4 Please amend your authorship list in your manuscript file to include author Emmanuel Daniel.

Response: Author Emmanuel Daniel is included in the revised manuscript.

5 Please amend the manuscript submission data (via Edit Submission) to include author Emmanuel Daniel Kija.

Response: Author Emmanuel Daniel Kija is omitted in the submission and in the manuscript.

6 . Please remove all personal information, ensure that the data shared are in accordance with participant consent, and re upload a fully anonymized data set. Note: spreadsheet columns with personal

Response: All person information is removed in the revised manuscript

7 Please include captions for your supporting information files at the end of your manuscript, and update any in-text citations to match accordingly. Please see our Supporting Information

Response: Thank you, caption for supporting information file is included in text citatio

REVIEWER #1: AUTHOR'S RESPONSES TO COMMENTS

S/N REVIEWER’S COMMENT AUTHOR’S RESPONSE

1 Why were the demographic information of the two participants interviewed to ensure data adequacy not reported? And were the interviews transcribed and analyzed? Please explain in detail and if not included in the analysis, amend this section of the results and explicitly state in the limitations section that further interviews were not conducted to ensure data adequacy after saturation was reached

Response: Thank you for your constructive comments. Amendments have been made to comply with your comment Page 10, 26 and 27

2 Add a column in Table 2 and report the open codes for each subtheme to facilitate tracking of findings and provide comprehensive findings for future researchers.

Response: Column in table 2 has been added to include open codes from participants quotes Page 13 -15

3 What is described in the text on line 403 for the number of subthemes in theme " " does not match the number of subthemes in the rest of the text and in Table 2. Please review and correct.

Response: Thank you for the constructive comment. The comment has been addressed in the revised manuscript Pages 21

4 Undoubtedly, the limitations of the study, especially methodological ones, include more issues that researchers need to pay attention to and carefully point out, including the use of only mothers' opinions and the failure to address the experiences of health professionals such as midwives and pediatricians who interacted with these mothers and infants and who have shared experiences. Meanwhile, triangulation was also created at the data source level, adding to the strength of the study

Response: The limitations of the study have been revised and now include the suggestion that the study relied on mothers' opinions and failed to account for the experiences of health professionals, such as midwives and paediatricians. Pages 26 and 27

REVIEWER #2: AUTHOR'S RESPONSES TO COMMENTS

S/N REVIEWER’S COMMENT AUTHOR’S RESPONSE

1 Please make the manuscript region-specific. There is a difference in the care provided in the high-, middle- and low-income countries.

• Add these parameters in the introduction, discussion and methods as well.

Response: Thank you for the constructive comment. The study explored more about lower-middle-income countries All manuscript

2 There is need to make the description of themes in a crisper and shortened format.

• The quotes of the patients may also be tabulated along with the themes

Response: Thanks for the comment, themes remained as it is short according to our view and open codes instead of patient quotes have been added in Table 2 to track findings, since patient codes may be tracked in the results Page 13-15

3. Our settings should be changed to a hospital setting. and Remove word semi semi-structured duplication

Response: Setting has been included in the revised manuscript, and the word semi has been omitted Page 2

4.Temperature fluctuation does not lead to asphyxia, and has to be deleted

Response: Changes have been made in the revised manuscript Page 3

5. This also shows there was a need to intervene appropriately by providing appropriate thermal care. This would be one of the ways to reduce NMR, not the only one.

Response: Thank you for the comment, the sentence has been revised and refined Page 3

6. What is the meaning of double increase rather than decline

Response: The meaning of double increase rather than decline means that the newborn mortality is still high compared to the Sustainable Development Goal of reducing newborn mortality to 12 per 1000 live births by 20230, as the newborn mortality rate in Tanzania is 24 per 1000 live births. Page 3

7. Ensure uniform citation formatting according to journal requirements

Response: All citations have been formatted in the revised manuscript

8. Strengthen the “knowledge gap” statement: explicitly indicate what is missing in existing literature from Tanzania/Kilimanjaro. Strengthening the “knowledge gap”

Response: Strengthen the “knowledge gap” statement is done in the revised manuscript Page 4

9. The rationale of the study being carried out in that particular region needs to be tailored according to the region and so needs modification in the wording

Response: Modification has been carried out to make it clear Page 4

10. The rationale given for the usage of this design may be avoided as it’s a known fact.

Response: The comment has been incorporated in the revised manuscript Page 5

11. Researchers chose the purposeful sampling technique to identify the population likely to possess detailed information on newborn hypothermia preventive practices” introduces a significant bias in the study. The mothers who know how to prevent in a detailed manner will be able to provide biased information.

Response: We appreciate your constructive feedback and had added limitation paragraph in the revised manuscript to clearly address this comment Page 26 and 27

12. Majority of the women were entrepreneur, employed. This also leads to bias. Describe strategies used to ensure maximum variation in sampling.

Response: Thank you for this valid concern about potential selection bias due to the overrepresentation of entrepreneurs and employed women. We incorporated strategies for maximum variation in our study to capture diverse socioeconomic and occupational perspectives through stratification of participants by contextual and key demographic characteristics to ensure heterogeneity beyond employment status, such as parity, education level, facility level, and experiences Page 6 and 12

13. Mention if the interview guide was pilot-tested or iteratively modified.

Response: Thank you for the valid comment, the tool was piloted and details has been included in revised manuscript Page 8

14. Add contextual details of the facilities(rural/urban, patient flow, type of maternity services). • Explain why these specific facilities were chosen

Response: Thanks for the comment, contextual details of the facilities has been added in the revised manuscript Page 6

15. Describe how coding disagreements were resolved.

Response: Coding disagreements were reviewed and discussed by the research team as explained in the revised manuscripts Page 10

15. Mention number of participants contributing to each theme (COREQ requirement).

Response: Thank you for the constructive comment. An explanation about the number of participants contributing to each theme is added in the revised manuscript Page 9

16. Add a short subsection on the study’s strengths (currently absent).

Response: Subsection of strength has been added in the current manuscripts Page 26

17. Provide a clearer articulation of how this work adds to regional evidence.

Response: Articulation of how this works adds to regional evidence has been added in the revised manuscript Page 26

18. References need rewriting.

Response: Thank you for the constructive feedback, all references have been formatted again in the revised manuscript Page 29,30,31 and 32

Attachment Submitted filename: Response to reviewers comments.docx

# Peer review: Decision Letter 1

**DOI:** 10.1371/journal.pone.0357756.r003

**Authors:** Syeda Humaida Hasan

4 Jun 2026

Dear Dr. Daniel,

Thank you for submitting your manuscript to PLOS ONE. After careful consideration, we feel that it has merit but does not fully meet PLOS ONE’s publication criteria as it currently stands. Therefore, we invite you to submit a revised version of the manuscript that addresses the points raised during the review process.

Please submit your revised manuscript by Jul 19 2026 11:59PM. If you will need more time than this to complete your revisions, please reply to this message or contact the journal office at plosone@plos.org. When you're ready to submit your revision, log on to https://www.editorialmanager.com/pone/ and select the 'Submissions Needing Revision' folder to locate your manuscript file.

- A letter that responds to each point raised by the academic editor and reviewer(s). You should upload this letter as a separate file labeled 'Response to Reviewers'.
- A marked-up copy of your manuscript that highlights changes made to the original version. You should upload this as a separate file labeled 'Revised Manuscript with Track Changes'.
- An unmarked version of your revised paper without tracked changes. You should upload this as a separate file labeled 'Manuscript'.

If you would like to make changes to your financial disclosure, please include your updated statement in your cover letter. Guidelines for resubmitting your figure files are available below the reviewer comments at the end of this letter.

If applicable, we recommend that you deposit your laboratory protocols in protocols.io to enhance the reproducibility of your results. Protocols.io assigns your protocol its own identifier (DOI) so that it can be cited independently in the future. For instructions see: https://journals.plos.org/plosone/s/submission-guidelines#loc-laboratory-protocols. Additionally, PLOS ONE offers an option for publishing peer-reviewed Lab Protocol articles, which describe protocols hosted on protocols.io. Read more information on sharing protocols at https://plos.org/protocols?utm_medium=editorial-email&utm_source=authorletters&utm_campaign=protocols.

As the corresponding author, your ORCID iD is verified in the submission system and will appear in the published article. PLOS supports the use of ORCID, and we encourage all coauthors to register for an ORCID iD and use it as well. Please encourage your coauthors to verify their ORCID iD within the submission system before final acceptance, as unverified ORCID iDs will not appear in the published article. *Only*  the individual author can complete the verification step; PLOS staff *cannot*  verify ORCID iDs on behalf of authors.

We look forward to receiving your revised manuscript.

Kind regards,

Syeda Humaida Hasan, Diploma in Child Health, FCPS (Pediatrics)

Academic Editor

PLOS One

Journal Requirements:

If the reviewer comments include a recommendation to cite specific previously published works, please review and evaluate these publications to determine whether they are relevant and should be cited. There is no requirement to cite these works unless the editor has indicated otherwise.

Please review your reference list to ensure that it is complete and correct. If you have cited papers that have been retracted, please include the rationale for doing so in the manuscript text, or remove these references and replace them with relevant current references. Any changes to the reference list should be mentioned in the rebuttal letter that accompanies your revised manuscript. If you need to cite a retracted article, indicate the article’s retracted status in the References list and also include a citation and full reference for the retraction notice.

[Note: HTML markup is below. Please do not edit.]

Reviewers' comments:

Reviewer's Responses to Questions

**Comments to the Author**

Reviewer #1: All comments have been addressed

Reviewer #2: All comments have been addressed

**********

2. Is the manuscript technically sound, and do the data support the conclusions?

Reviewer #1: Yes

Reviewer #2: Yes

**********

3. Has the statistical analysis been performed appropriately and rigorously?

Reviewer #1: Yes

Reviewer #2: Yes

**********

4. Have the authors made all data underlying the findings in their manuscript fully available?

The PLOS Data policy

Reviewer #1: Yes

Reviewer #2: Yes

**********

5. Is the manuscript presented in an intelligible fashion and written in standard English?

Reviewer #1: No

Reviewer #2: No

**********

Reviewer #1: Dear Author,

The article is well written and somewhat detailed and can provide valuable knowledge for future studies in neonatal health programs. Minor suggestions for improving the article are provided in the attached file. Among the suggestions are as follows:

Abstract

Method:

The number of participants in the method should be added and removed from the method.

Conclusion:

The conclusion is not based on the findings. On what basis did you identify the need for education for mothers? It is suggested that it be revised.

Introduction:

This section was well written, the importance and to an acceptable extent the necessity of conducting the study was described. I appreciate that it was not long and that the importance and necessity of conducting the study were comprehensively addressed.

Method:

The number of participants should be reported.

Reviewer #2: thank you for the review.

The English language may be made simpler for the wider audience. Esoteric words may be decreased. It will help the people in LMIC to understand and disseminate it better.

**********

what does this mean?). If published, this will include your full peer review and any attached files.

If you choose “no”, your identity will remain anonymous but your review may still be made public.

**Do you want your identity to be public for this peer review?**  For information about this choice, including consent withdrawal, please see our Privacy Policy

Reviewer #1: **Yes:**  Mehrsadat Mahdizadeh

Reviewer #2: No

**********

[NOTE: If reviewer comments were submitted as an attachment file, they will be attached to this email and accessible via the submission site. Please log into your account, locate the manuscript record, and check for the action link "View Attachments". If this link does not appear, there are no attachment files.]

To ensure your figures meet our technical requirements, please review our figure guidelines: https://journals.plos.org/plosone/s/figures

You may also use PLOS’s free figure tool, NAAS, to help you prepare publication quality figures: https://journals.plos.org/plosone/s/figures#loc-tools-for-figure-preparation.

NAAS will assess whether your figures meet our technical requirements by comparing each figure against our figure specifications.

Attachment Submitted filename: PONE-D-25-62885_R1_reviewer.pdf

# Author comment: Author response to Decision Letter 2

**DOI:** 10.1371/journal.pone.0357756.r004

6 Jun 2026

REVIEWER 1

Comment: The number of participants in the method should be added and removed from the method

Response: Thank you for your constructive suggestion. The number of participants has been included in the method section

Comment: The conclusion is not based on the findings. On what basis did you identify the need for education for mothers? It is suggested that it be revised.

Response: Thanks for the comment, conclusion has been improved based on findings in the revised manuscript

Comment: The number of participants should be reported on the method section

Response: Thank you for the comments, participants have been added in the method section in the revised manuscript

REVIEWER 2

Comment: The English language may be made simpler for the wider audience. Esoteric words may be decreased. It will help the people in LMIC to understand and disseminate it better.

Response: Thank you for the constructive comments. The author(s) have used simple English and understandable even in LMIC

Attachment Submitted filename: Response to reviewers 1 &2 comments.docx

# Peer review: Decision Letter 2

**DOI:** 10.1371/journal.pone.0357756.r005

**Authors:** Syeda Humaida Hasan

2 Jul 2026

Dear Dr. Daniel,

Thank you for submitting your manuscript to PLOS ONE. After careful consideration, we feel that it has merit but does not fully meet PLOS ONE’s publication criteria as it currently stands. Therefore, we invite you to submit a revised version of the manuscript that addresses the points raised during the review process.

==============================

Check grammar:

Line 48-49 These findings call for comprehensive 49 promoting a positive behaviour on thermal newborn protection.

Improper capilization in line 57: Moderate hypothermia

Make it simple sentence line 60-63

According to the World Health Organisation (WHO), it estimated that 2.3 million newborns died in 2022 within 28 days of life, whereby SubSaharan countries had the highest newborn mortality in the world at 27 deaths per 1000 live births .

Improper spacing in line 64

Please clarify the correct phrase: epistemological position or ability

(Unlike more 96 abstract methodologies, this design was selected for its epistemological ability to provide a thinly 97 theorized yet high-fidelity account of participant’s lived experiences.)

272. table. Check spelling of ‘bag’

**General Comments**

Lines 48–49: Please revise the sentence, *"These findings call for comprehensive promoting a positive behaviour on thermal newborn protection."*  The sentence is grammatically incorrect and should be rewritten for clarity.

Line 57**:**  Please correct the capitalization of moderate hypothermia.

Lines 60–63: Please simplify the following sentence to improve readability:

*"According to the World Health Organization (WHO), it was estimated that 2.3 million newborns died within the first 28 days of life in 2022, with sub-Saharan Africa having the highest neonatal mortality rate in the world at 27 deaths per 1,000 live births."*

Line 64: Please check and correct the spacing.

Lines 95–97: Please clarify the terminology. Is **"** epistemological position**"**  intended rather than "epistemological ability"?

*"Unlike more abstract methodologies, this design was selected for its epistemological ability to provide a thinly theorized yet high-fidelity account of participants' lived experiences."*

Table (around Line 272): Please check the spelling of **"** bag."

Line 344: Please check the spelling of “soup”

**Abstract – Conclusion**

The conclusion is not fully aligned with the study methodology. As this is a qualitative descriptive study based on interviews with 12 postnatal mothers, the findings describe mothers' reported thermal care practices rather than evaluate the effectiveness of these practices in preventing neonatal hypothermia. Therefore, statements such as **"** significantly reduces the risk of newborn hypothermia" and identifying skin-to-skin contact as the "cornerstone intervention" are stronger than the evidence generated by this study can support. The conclusion should instead emphasize that these practices were commonly reported by participants and may inform educational interventions or future quantitative studies evaluating their effectiveness.

**Methods**

The methods section includes most of the essential components of a qualitative study; however, several methodological inconsistencies and reporting gaps remain. The manuscript continues to mix concepts from different qualitative traditions, for example by referring to "phenomenological integrity" despite describing the study as a qualitative descriptive design.

Please consider whether concepts such as phenomenological integrity, prolonged engagement, intercoder reliability, and transferability are appropriate for this study design. If these concepts are retained, their use should be clearly justified and aligned with the stated qualitative methodology. Otherwise, they should be replaced with terminology that is consistent with a qualitative descriptive approach.

Descriptions of the audio recorder's technical features are unnecessarily detailed and should be replaced with information that is methodologically relevant.

Several procedures also require further clarification, including the forward–backward translation process, participant recruitment, transcript verification (member checking), assessment of data saturation.

==========================

Please submit your revised manuscript by Aug 16 2026 11:59PM. If you will need more time than this to complete your revisions, please reply to this message or contact the journal office at plosone@plos.org. When you're ready to submit your revision, log on to https://www.editorialmanager.com/pone/ and select the 'Submissions Needing Revision' folder to locate your manuscript file.

- A letter that responds to each point raised by the academic editor and reviewer(s). You should upload this letter as a separate file labeled 'Response to Reviewers'.
- A marked-up copy of your manuscript that highlights changes made to the original version. You should upload this as a separate file labeled 'Revised Manuscript with Track Changes'.
- An unmarked version of your revised paper without tracked changes. You should upload this as a separate file labeled 'Manuscript'.

If you would like to make changes to your financial disclosure, please include your updated statement in your cover letter. Guidelines for resubmitting your figure files are available below the reviewer comments at the end of this letter.

If applicable, we recommend that you deposit your laboratory protocols in protocols.io to enhance the reproducibility of your results. Protocols.io assigns your protocol its own identifier (DOI) so that it can be cited independently in the future. For instructions see: https://journals.plos.org/plosone/s/submission-guidelines#loc-laboratory-protocols. Additionally, PLOS ONE offers an option for publishing peer-reviewed Lab Protocol articles, which describe protocols hosted on protocols.io. Read more information on sharing protocols at https://plos.org/protocols?utm_medium=editorial-email&utm_source=authorletters&utm_campaign=protocols.

As the corresponding author, your ORCID iD is verified in the submission system and will appear in the published article. PLOS supports the use of ORCID, and we encourage all coauthors to register for an ORCID iD and use it as well. Please encourage your coauthors to verify their ORCID iD within the submission system before final acceptance, as unverified ORCID iDs will not appear in the published article. *Only*  the individual author can complete the verification step; PLOS staff *cannot*  verify ORCID iDs on behalf of authors.

We look forward to receiving your revised manuscript.

Kind regards,

Syeda Humaida Hasan, Diploma in Child Health, FCPS (Pediatrics)

Academic Editor

PLOS One

**Journal Requirements:**

1. If the reviewer comments include a recommendation to cite specific previously published works, please review and evaluate these publications to determine whether they are relevant and should be cited. There is no requirement to cite these works unless the editor has indicated otherwise.

2. Please review your reference list to ensure that it is complete and correct. If you have cited papers that have been retracted, please include the rationale for doing so in the manuscript text, or remove these references and replace them with relevant current references. Any changes to the reference list should be mentioned in the rebuttal letter that accompanies your revised manuscript. If you need to cite a retracted article, indicate the article’s retracted status in the References list and also include a citation and full reference for the retraction notice.

[Note: HTML markup is below. Please do not edit.]

Reviewers' comments:

Reviewer's Responses to Questions

**Comments to the Author**

Reviewer #1: All comments have been addressed

Reviewer #2: All comments have been addressed

**********

2. Is the manuscript technically sound, and do the data support the conclusions?

Reviewer #1: Yes

Reviewer #2: Yes

**********

3. Has the statistical analysis been performed appropriately and rigorously?

Reviewer #1: N/A

Reviewer #2: Yes

**********

4. Have the authors made all data underlying the findings in their manuscript fully available?

The PLOS Data policy

Reviewer #1: Yes

Reviewer #2: Yes

**********

5. Is the manuscript presented in an intelligible fashion and written in standard English?

Reviewer #1: Yes

Reviewer #2: Yes

**********

Reviewer #1: Dear Author

I thank you for your efforts in improving the manuscript, which provides valuable results, in accordance with the suggestions of the esteemed reviewers.

Reviewer #2: I congratulate the authors for addressing all the comments and carrying out a very relevant study in the low middle income countries.

**********

what does this mean?). If published, this will include your full peer review and any attached files.

If you choose “no”, your identity will remain anonymous but your review may still be made public.

**Do you want your identity to be public for this peer review?**  For information about this choice, including consent withdrawal, please see our Privacy Policy

Reviewer #1: **Yes:**  Mehrsadat Mahdizadeh

Reviewer #2: No

**********

[NOTE: If reviewer comments were submitted as an attachment file, they will be attached to this email and accessible via the submission site. Please log into your account, locate the manuscript record, and check for the action link "View Attachments". If this link does not appear, there are no attachment files.]

To ensure your figures meet our technical requirements, please review our figure guidelines: https://journals.plos.org/plosone/s/figures

You may also use PLOS’s free figure tool, NAAS, to help you prepare publication quality figures: https://journals.plos.org/plosone/s/figures#loc-tools-for-figure-preparation.

NAAS will assess whether your figures meet our technical requirements by comparing each figure against our figure specifications.

Attachment Submitted filename: PONE-D-25-62885_R2_reviewer.pdf

# Author comment: Author response to Decision Letter 3

**DOI:** 10.1371/journal.pone.0357756.r006

16 Aug 2026

Comment: Check grammar: Line 48-49 These findings call for comprehensive 49 promoting a positive behaviour on thermal newborn protection.

Response: Thank you for the constructive comment, we have worked to correct grammar in the revised manuscript Page 2-3

Comment: Improper capilization in line 57: Moderate hypothermia

Response: Thank you, Moderate hypothermia has been corrected to moderate hypothermia in the revised manuscript Page 3-line 54

Comment: Make it simple sentence line 60-63

Response: Thanks, stipulated sentence has been made simple Page 3

Comment: Please clarify the correct phrase: epistemological position or ability

Response: Thank you, the study approach, and design was reviewed again and written well in the revised manuscript Page 5

Comment: 272. table. Check spelling of ‘bag’

Response: Thanks, spelling for bag was checked and corrected Page 14

Comment: Line 344: Please check the spelling of “soup”

Response: Thank you for the constructive comment, the spelling of soup is correct which mean that soup is a liquid food especially with meat, fish or vegetable stock as a base and often containing pieces of solid food- according to medical dictionary Page 18

Comment: The conclusion is not fully aligned with the study methodology.

Response: Thank you for the comment, we have corrected our conclusion to align with our study methodology as suggested Page 3

Comment: Please consider whether concepts such as phenomenological integrity, prolonged engagement, intercoder reliability, and transferability are appropriate for this study design. If these concepts are retained, their use should be clearly justified and aligned with the stated qualitative methodology. Otherwise, they should be replaced with terminology that is consistent with a qualitative descriptive approach.

Response: Thank you, concepts such as phenomenological integrity, prolonged engagement, intercoder reliability, and transferability have been revised to its applicability and the method section has been revised again to align with qualitative descriptive approach Page 5-11

Comment: Descriptions of the audio recorder's technical features are unnecessarily detailed and should be replaced with information that is methodologically relevant.

Response: Thanks, your suggestion has been put into consideration. Description of audio recorders were removed in the revised manuscript Page 8

Comment: Several procedures also require further clarification, including the forward–backward translation process, participant recruitment, transcript verification (member checking), assessment of data saturation.

Response: Thank you for the comment, a thorough clarification of forward backward translation process, participants recruitment, member checking and assessment of data saturation has been made for easy understanding, Page 7,9 and 11

Attachment Submitted filename: Response to reviewers comments.docx

# Editor report: Decision Letter 3

**DOI:** 10.1371/journal.pone.0357756.r007

**Authors:** Syeda Humaida Hasan

23 Aug 2026

Exploring preventive practices of newborn hypothermia among post-natal mothers in selected public health facilities in Kilimanjaro region: Qualitative descriptive study

PONE-D-25-62885R3

Dear Dr. Daniel,

We’re pleased to inform you that your manuscript has been judged scientifically suitable for publication and will be formally accepted for publication once it meets all outstanding technical requirements.

Within one week, you’ll receive an e-mail detailing the required amendments. When these have been addressed, you’ll receive a formal acceptance letter and your manuscript will be scheduled for publication.

An invoice will be generated when your article is formally accepted. Please note, if your institution has a publishing partnership with PLOS and your article meets the relevant criteria, all or part of your publication costs will be covered. Please make sure your user information is up-to-date by logging into Editorial Manager at Editorial Manager® and clicking the ‘Update My Information' link at the top of the page. For questions related to billing, please contact billing support.

If your institution or institutions have a press office, please notify them about your upcoming paper to help maximize its impact. If they’ll be preparing press materials, please inform our press team as soon as possible -- no later than 48 hours after receiving the formal acceptance. Your manuscript will remain under strict press embargo until 2 pm Eastern Time on the date of publication. For more information, please contact onepress@plos.org.

Kind regards,

Syeda Humaida Hasan, Diploma in Child Health, FCPS (Pediatrics)

Academic Editor

PLOS One

Additional Editor Comments (optional):

Reviewers' comments:

# Editor report: Acceptance letter

**DOI:** 10.1371/journal.pone.0357756.r008

**Authors:** Syeda Humaida Hasan

PONE-D-25-62885R3

PLOS One

Dear Dr. Daniel,

I'm pleased to inform you that your manuscript has been deemed suitable for publication in PLOS One. Congratulations! Your manuscript is now being handed over to our production team.

At this stage, our production department will prepare your paper for publication. This includes ensuring the following:

* All references, tables, and figures are properly cited

* All relevant supporting information is included in the manuscript submission,

* There are no issues that prevent the paper from being properly typeset

You will receive further instructions from the production team, including instructions on how to review your proof when it is ready. Please keep in mind that we are working through a large volume of accepted articles, so please give us a few days to review your paper and let you know the next and final steps.

Lastly, if your institution or institutions have a press office, please let them know about your upcoming paper now to help maximize its impact. If they'll be preparing press materials, please inform our press team within the next 48 hours. Your manuscript will remain under strict press embargo until 2 pm Eastern Time on the date of publication. For more information, please contact onepress@plos.org.

You will receive an invoice from PLOS for your publication fee after your manuscript has reached the completed accept phase. If you receive an email requesting payment before acceptance or for any other service, this may be a phishing scheme. Learn how to identify phishing emails and protect your accounts at https://explore.plos.org/phishing.

If we can help with anything else, please email us at customercare@plos.org.

Thank you for submitting your work to PLOS One and supporting open access.

Kind regards,

PLOS One Editorial Office Staff

on behalf of

Dr. Syeda Humaida Hasan

Academic Editor

PLOS One
